# Synergistic Interplay between Intermolecular Halogen and Chalcogen Bonds in the Dihalogen Adducts of 2,5-Bis(pyridine-2-yl)tellurophene: Reactivity Insights and Structural Trends

**DOI:** 10.1021/acs.inorgchem.5c01084

**Published:** 2025-05-29

**Authors:** Enrico Podda, Massimiliano Arca, Maria Carla Aragoni, Claudia Caltagirone, Vito Lippolis, Anna Pintus, Douglas B. Paixão, Eduardo G. O. Soares, Paulo H. Schneider

**Affiliations:** † Centro Servizi di Ateneo per la Ricerca (CeSAR), 3111 Università degli Studi di Cagliari, S.S. 554 Bivio per Sestu, Monserrato, CA 09042, Italy; ‡ Dipartimento di Scienze Chimiche e Geologiche, 96940 Università degli Studi di Cagliari, S.S. 554 Bivio per Sestu, Monserrato, CA 09042, Italy; § Institute of Chemistry, 28124 Federal University of Rio Grande do Sul (UFRGS), Av. Bento Gonçalves 9500 P.O. Box: 15003, Porto Alegre, RS 91501-970, Brazil

## Abstract

The reactivity of 2,5-bis­(pyridine-2-yl)­tellurophene (**L**) toward elemental dihalogens XY (X = Y = I, Br; X = I, Y = Cl, Br) was explored. The oxidative addition of the dihalogen molecules to the Te­(II) center was observed in **L**I_2_ (**1**), **L**I_2_·1/2I_2_ (**2**), **L**Br_2_ (**3**), **L**Br_1.63_I_0.37_ (**4**), and **L**Cl_1.86_I_0.14_ (**5**), which were characterized in the solid state by X-ray diffraction analysis and Raman spectroscopy. In all cases, a seesaw geometry at the chalcogen atom was observed with the linear X−Te−X moiety almost perpendicular to the tellurophene ring. The crystal packing in these compounds displays a peculiar and synergistic interplay of halogen and chalcogen bonds. A comparison with analogous compounds reported in the literature was carried out to establish the key factors determining the supramolecular arrays of noncovalent intermolecular interactions (NCIs) observed in this class of compounds.

## Graphical abstract

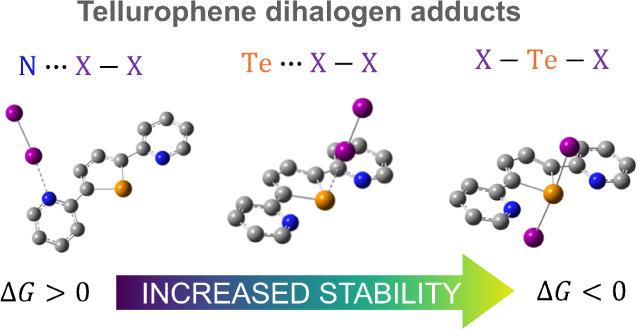

## Introduction

Recent years have seen a growing interest in categorizing and understanding the nature of directional intermolecular noncovalent interactions (NCIs) of variable strength, such as hydrogen bonds (HBs),^1–3^ halogen bonds (HaBs),^4–9^ and chalcogen bonds (ChBs),^10–16^ among others. The competition and cooperativity among these interactions play a significant role in determining the crystal structure and supramolecular architecture of crystalline materials built from molecular synthons containing halogen and chalcogen atoms in their skeletons.^17–26^ The great attention to these interactions and the factors affecting their strength and directionality is primarily due to the robustness of the resulting supramolecular architectures, and to the theoretical possibility of predicting their topology to design new crystalline materials and thus control their physical properties.^27–31^

A comparative analysis of crystal structures, especially within a family of related compounds, is a valuable approach for investigating how specific molecular tectons tend to form particular supramolecular architectures. It helps elucidate how subtle changes in the molecular backbone can affect the geometry and hierarchy of intermolecular interactions and ultimately the resulting crystal structure. Recent studies have explored this area, some using specialized automated software to identify structural similarities.^32–34^

In this respect, some classes of products obtainable from the reaction between organochalcogen­(II) compounds (RCh, R_2_Ch; R = organic framework; Ch = S, Se, Te) and elemental dihalogens (XY, X = Y = I, Br, Cl; X = I, Y = Cl, Br) represent ideal study cases.^35–37^ These reactions can follow a variety of pathways depending on the experimental conditions used, such as the polarity of the solvent, the reaction molar ratio, and the acid−base nature of the reactants, including their structural features and the nature of the chalcogen atom involved. The most notable pathways include the formation of neutral charge-transfer (CT) “spoke” adducts, which feature an almost linear Ch−X−Y moiety (and formally a HaB),^38–49^ and “T-shaped” (TS) or seesaw adducts (depending on the nature of the organochalcogen­(II) compound), resulting from the oxidative addition of a dihalogen molecule to the chalcogen atom, and containing a X−Ch−Y fragment (and formally a ChB), with the chalcogen atom in the oxidation state IV.^50–60^ Other different structural archetypes have also been established by X-ray diffraction analysis and vibrational spectroscopy (especially FT-Raman) for the products of these reactions, particularly with organochalcogenone donors (RCh). They mainly include two-chalcogen-coordinated halogen­(I) complexes ([RCh−X−ChR]^+^) and cations containing a chalcogen−chalcogen single bond ([RCh−ChR]^*n*+^, *n* = 1, 2), balanced by discrete or extended polyhalides.^35,43,60–64^ These compounds and their formation pathways have attracted much interest, due to their potential relevance to material chemistry, biology and pharmacological activities, and particularly for their involvement in the mechanism of action of drugs used in the treatment of hyperthyroidism.^64–68^

Predicting the outcome of the reactions between organochalcogen­(II) compounds and elemental dihalogens is still a challenging task, although the Natural Bond Order (NBO) charge distribution^69^ on the hypothetical [RCh−X]^+^ cation intermediate allegedly formed in solution^55,70,71^ can be of great help.^63,70,72,73^ A general broad qualitative observation is that on decreasing the electronegativity difference between the halogen X and the chalcogen species Ch, CT-adducts are more likely formed as compared to TS or seesaw adducts.^74^ Indeed, the number of structurally characterized insertion adducts generally decreases on passing from Cl_2_ to Br_2_ and I_2_ for organosulfur­(II) and organoselenium­(II) donor molecules, and sulfur insertion adducts with I_2_ are unknown. On the other hand, to the best of our knowledge, only two CT I_2_-adducts are known for organic compounds containing organotellurium­(II) as the donor atom.^48,75^ By limiting the discussion to I_2_ (the less electronegative and oxidizing among dihalogens), while aliphatic organochalcogen­(II) donors (R_2_Ch) generally afford CT-adducts, chalcogeno­(II)-amides (RCh) (especially imidazoline-2-chalcogenone derivatives, Ch = S, Se) can also give rise to the formation of TS-adducts and the other oxidation products (see above).^60,63^ Apart from very few exceptions,^41,47,60,76,77^ this tendency is the rule when considering the reactions with Br_2_, especially with organotellurium­(II) compounds,^78–80^ which are prone to undergo dihalogen oxidative addition even with I_2_,^58^ due to the metalloid character of tellurium and its facility to shuttle between Te­(II) and Te­(IV) and assume different coordination numbers.

Although the CT and insertion adducts of chalcogen­(II) donor molecules with elemental dihalogens are characterized by distinctive molecular structural features, the nature of the chalcogen atom, the molecular shape, and the nature of the dihalogen involved can finely tune the polarization of the Ch−X and X−Y bonds within the Ch−X−Y and X−Ch−Y fragments. This, in turn, affects the strength and directionality of the intermolecular HaB and ChB interactions, eventually influencing their interplay in determining the supramolecular architecture and crystal packing features in these compounds.

Among the great variety and structural diversity of chalcogen­(II) donor molecules, chalcogenophenes—particularly tellurophene-based small molecules and polymers—have attracted significant interest due to their unique redox and photophysical properties, as well as their optoelectronic applications in organic photovoltaics (OPVs), organic field effect transistors (OFETs), and sensors.^55,56,81–84^

Although the oxidative addition and thermal/photoreductive elimination of Br_2_ has been deeply investigated for π-conjugated tellurophenes in the context of the development of new photochemically active compounds supported by main group elements,^55,56^ the supramolecular structural chemistry of the resulting insertion adducts, as well as the interplay between the chalcogen−halogen and halogen−halogen interactions when varying the halogen and substituents in the tellurophene skeleton, has not yet been considered.

In this paper, we report on the reactivity of 2,5-bis­(pyridine-2-yl)­tellurophene (**L**) with I_2_, Br_2_, IBr, and ICl and the analysis of the resulting products in the solid state in comparison with analogous compounds reported in the literature with the π-conjugated 2,5-disubstitutedtellurophenes **L** ^**a**^−**L** ^**f**^ (Scheme 1).^55,56,85–87^

**Scheme 1:**
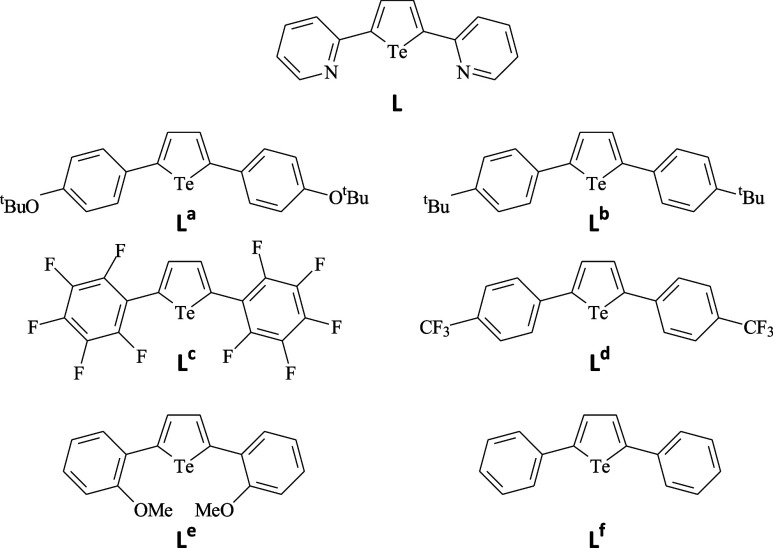
Schematic Drawings of 2,5-Bis­(pyridine-2-yl)­tellurophene (**L**) Considered in This Paper, along with Analogous π-Conjugated Tellurophenes Whose Reactions with Elemental Dihalogens Were Previously Reported^55,56,85–87^

With respect to **L** ^**a**^−**L** ^**f**^, **L** is also characterized by the presence of two nitrogen-heterocycle donor sites, which could potentially interact with the dihalogen molecules or affect both the reactivity of the tellurium­(II) center and the intermolecular interactions in the crystal packing of the resulting products.

## Results and Discussion

### Synthesis and Structural Analysis

2,5-Bis­(pyridine-2-yl)­tellurophene (**L**) was synthesized by reacting 1,4-bis­(pyridine-2-yl)-1,3-butadiyne with Te^2−^ generated *in situ* from elemental Te^0^ by using rongalite in PEG-400 as an efficient, inexpensive, and environmentally friendly reductive system.^87^ An X-ray diffraction analysis was undertaken on single crystals of **L** grown from a CHCl_3_ solution. The asymmetric unit consists of two planar crystallographically independent molecules of **L** (Tables S1, S2 and Figure S1) in a periplanar conformation with the aromatic N donors disposed on the same side of the tellurium­(II) center. The crystal packing is determined by π−π stacking interactions between the pyridyl groups of symmetry-related units of **L** (Figure S2).

**L** was made to react with I_2_, Br_2_, ICl, and IBr, and the crystals formed by partial slow evaporation or cooling of the resulting solutions, according to Scheme 2, were isolated and analyzed in the solid state by X-ray diffraction and Raman spectroscopy.

**Scheme 2:**
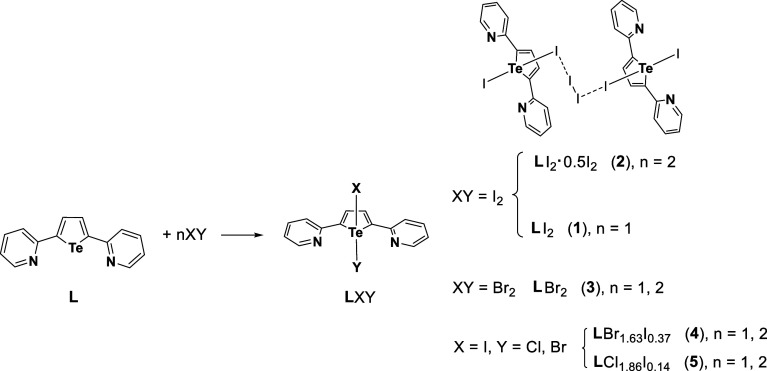
Outline of the Compounds Reported in the Present Study from the Structural Characterization

The reaction of **L** with I_2_ in 1:1 molar ratio in CHCl_3_ afforded after 72 h at −20 °C of the reaction mixture, red block-shaped crystals corresponding to the formulation **L**I_2_ (**1**). The crystal structure confirms the oxidative addition of I_2_ to the tellurium­(II) atom of **L** (Tables S1, S3 and Figure 1), as the first case of an I_2_ adduct of this type for a tellurophene system.

**Figure 1:**
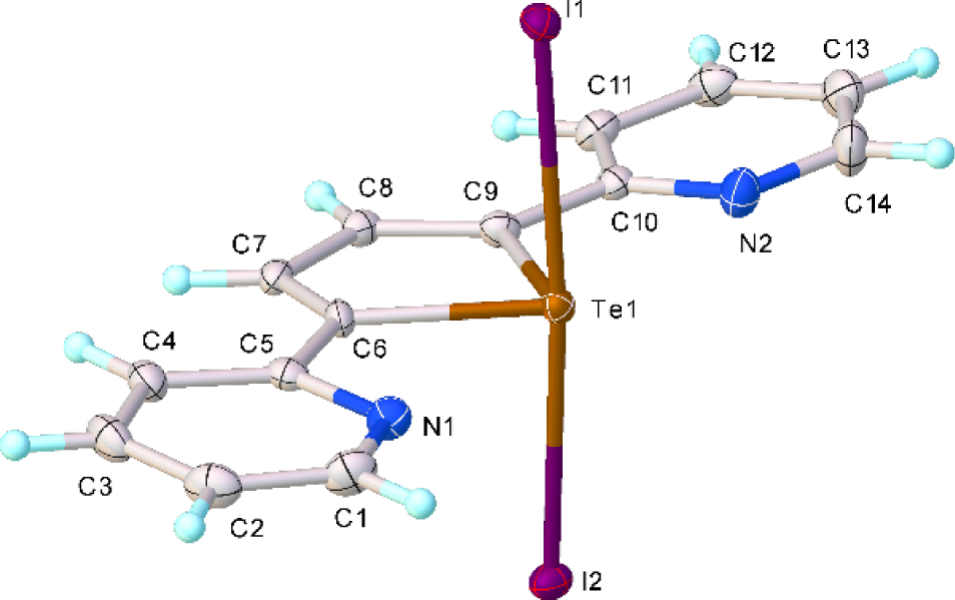
X-ray crystal structure of **L**I_2_ (**1**) showing the atom labeling scheme adopted. Thermal ellipsoids are drawn at 50% probability level.

The asymmetric unit consists of a single adduct molecule in which both pyridyl rings adopt a periplanar conformation with respect to the central tellurophene moiety. A seesaw geometry can be observed at the tellurium­(IV) atom [Te1−I1 = 2.9223(4), Te1−I2 = 2.8961(4) Å; I1−Te1−I2 = 174.244(8)°]. This geometry arises from the stereochemically active lone pair (LP) of electrons at the Te atom: the chalcogen atom adopts a distorted trigonal bipyramid geometry in which the three equatorial positions are occupied by the carbon atoms and the LP, and the I atoms occupy the axial positions. **L**I_2_ units interact with each other via Te···I, I···I, and CH···I intermolecular interactions to afford a 2D assembly (Figure 2).

**Figure 2:**
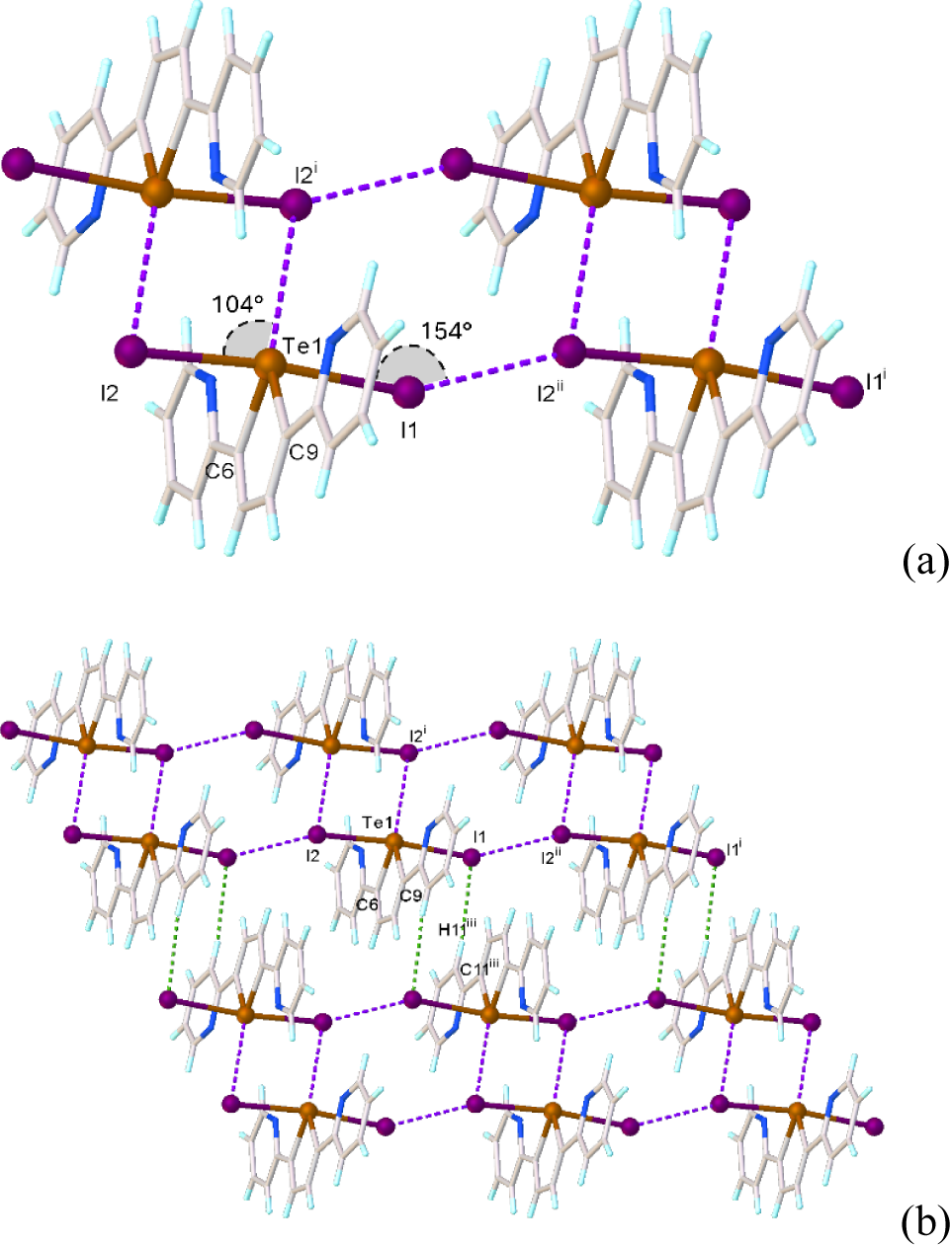
Intermolecular interactions in the crystal structure of **L**I_2_ (**1**) (a); partial view of the crystal packing of **1** along the *b*-axis (b). Symmetry codes: ^i^ = 1−*x*, 1−*y*, and −*z*; ^ii^ = 1+*x*, +*y*, and +*z*; ^iii^ = 2−*x*, 2−*y*, and 1−*z*.

In particular, couples of symmetry-related adduct units establish weak Te···I intermolecular interactions with an interatomic distance of 3.9910(4) Å (Te1···I2^i^ in Figure 2; ^i^ = 1−*x*, 1−*y*, and −*z*), which are clearly below the sum of the van der Waals radii (Σ*R*_vdW_ = 4.2 Å) and above the sum of the covalent radii (Σ*R*_cov_ = 2.77 Å), resulting in *pseudo*-square Te_2_I_2_ motifs [I2−Te1−I2^i^ 104.12(4)°]. Dimers of this type interact with each other via I···I interactions with an interatomic distance of 3.7171(4) Å (I1···I2^ii^ in Figure 2; ^ii^ = 1+*x*, +*y*, +*z*) to afford ladder-like ribbons propagating parallel to the *a*-axis [Te1−I1−I2^ii^ = 154.19 (3)°]. Weak CH···I contacts (H11^iii^···I1 = 3.08 Å; C11^iii^−H11^iii^···I1 = 173°; ^iii^ = 2−*x*, 2−*y*, 1−*z*) and slipped π−π stacking interactions between pyridyl rings interconnect the ribbons in a 2D assembly (Figure S3).

According to Desiraju et al.,^88^ the I···I interactions responsible for the formation of the ladder-like ribbons can be classified as type I HaBs with identical Te−I···I angles. These interactions are quite common in R_2_TeI_2_ compounds^23^ and for the explanations of their packing effects in the crystals, anisotropic effects between elliptically shaped halogen atoms and dispersion contributions have been invoked.^23,88^

As far as the weak Te···I intermolecular interactions responsible for the dimeric association of the **L**I_2_ units, they are more difficult to categorize, as they do not fully follow the geometry expected for a typical σ-hole interaction (either HaB or ChB). In fact, the negative belt of the iodine atom from one **L**I_2_ unit appears to point toward the Te atom of the other unit, thus behaving as charge donating atom (nucleophile), with a Te−I−Te angle quite far from linearity. This is not consistent with the formation of a HaB of type II (σ-hole interactions are named by the atom interacting at the region of positive electrostatic potential, i.e., the electrophile). On the other hand, the negative belt of the iodine atom does not point exactly toward either one of the two directions defined by the two C−Te bonds [C9−Te1−I2^i^ = 160.39 (2)°, C6−Te1−I2^i^ = 115.22 (6)°], where the two σ-holes on the tellurium atom are expected to be present, but rather seems to be shifted toward the lone pair on the chalcogen atom. This is also not consistent with the formation of a classical ChB, for which a C−Te−I angle close to 180° should be observed. More insights into these Te···I infringing contacts will be provided in the theoretical section.

The reaction of **L** with I_2_ in CHCl_3_ was also performed with a 1:2 (**L**/I_2_) reagent molar ratio. Orange needle-shaped crystals were obtained upon the slow evaporation of the solvent at room temperature. X-ray diffraction analysis revealed the presence in the asymmetric unit of a seesaw **L**I_2_ adduct deriving from the oxidative addition of I_2_ to **L** and half of a cocrystallized I_2_ molecules lying on a crystallographic inversion center (Tables S1, S4 and Figure 3a), consistent with a formulation **L**I_2_·1/2I_2_ (**2**) for the compound. The Te−I bond lengths in the **L**I_2_ tellurium­(IV) adduct unit are slightly asymmetric [Te1−I1 = 2.8750(12), Te1−I2 = 2.9511(12) Å; I1−Te1−I2 = 175.39(4)°] due to the interaction with the cocrystallized I_2_ molecule [I3−I3^i^ = 2.7326(18) Å]. As a result, in **2**, the cocrystallized diiodine molecule bridges via type II HaBs^88^ two **L**I_2_ units, affording a “Z-shaped” I−Te−I···I−I···I−Te−I (Te_2_I_6_) motif as shown in Figure 3b [I2−I3 = 3.4553(13) Å; I2−I3−I3^i^ = 178.03(6)°, Te1−I2−I3 107.04(3)°; ^i^ = 1−*x*, 2−*y*, −*z*].

**Figure 3:**
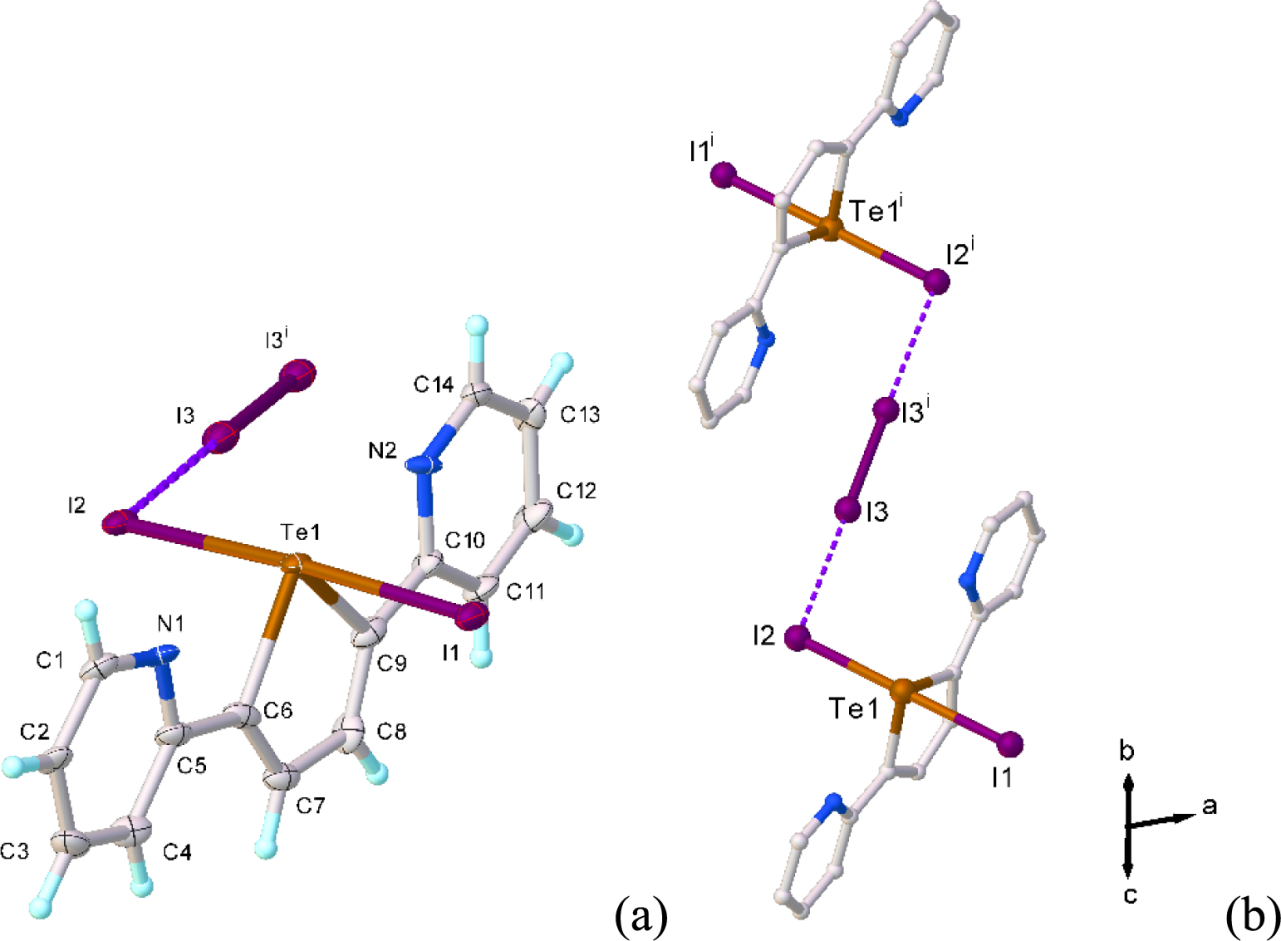
X-ray crystal structure of **L**I_2_·1/2I_2_ (**2**) showing the atom labeling scheme adopted (a). Thermal ellipsoids are drawn at 50% probability level. View of the “Z-shaped” **L**I_2_···I_2_···**L**I_2_ motif in **2** (b). Symmetry codes: ^i^ = 1−*x*, 2−*y*, and −*z*.

Analogously to **1**, also in **2** both pyridyl rings adopt a periplanar conformation with respect to the central tellurophene moiety, with the two planar π-conjugated organic moieties related by inversion symmetry and disposed on opposite sides of the plane containing the “Z-shaped” Te_2_I_6_ backbone (Figure 3).

Discrete **D**X_2_···X_2_···**D**X_2_ motifs similar to that observed in **2**, or infinite ···(**D**X_2_···X_2_···**D**X_2_)_∞_··· chain-like assemblies (in the case where both halides at the chalcogen­(IV) center are involved in type II HaBs with cocrystallized dihalogen molecules, **D**X_2_ = adduct from the oxidative addition of a dihalogen molecule to an organochalcogen­(II) compound), although documented in the literature, are quite rare.^54,89–94^

In the case of the reaction between 1,3-diethyl-4,5-dimethyl-imidazoline-2-selone (**D**) and elemental diiodine resulting in the compound of formulation **D**I_2_·1/2I_2_, the interaction of I_2_ with the I−Se−I moiety of the TS I_2_-adduct induces such a high polarization of the chalcogen−halogen bond within the insertion **D**I_2_ adduct that the system is better described as ···{[I-(R)­Se]^+^···(I_3_)^−^···[I-(R)­Se]^+^}_∞_··· infinite chains of alternating and interacting head-to-tail [I-(R)­Se]^+^ cation and I_3_^−^ anions, held by ChBs and type II HaBs.^92^

“Z-shaped” **L**I_2_···I_2_···**L**I_2_ motifs in **2** are assembled in ribbons parallel to the *c*-axis via Te···I interactions similar in directionality to those observed in **1** forming *pseudo*-square Te_2_I_2_ motifs, but slightly shorter (Te1···I2^ii^ = 3.9093 (13) Å in Figure 4a; C9−Te1−I2^ii^ = 114.0(4), C6−Te1−I2^ii^ = 162.2(4)°, ^ii^ = 1−*x*, 2−*y*, 1−*z*). In the crystal packing, ribbons of this kind associate via π−π stacking interactions between symmetry-related pyridyl rings (intercentroid distance = 3.49 Å, Figure 4b).

**Figure 4:**
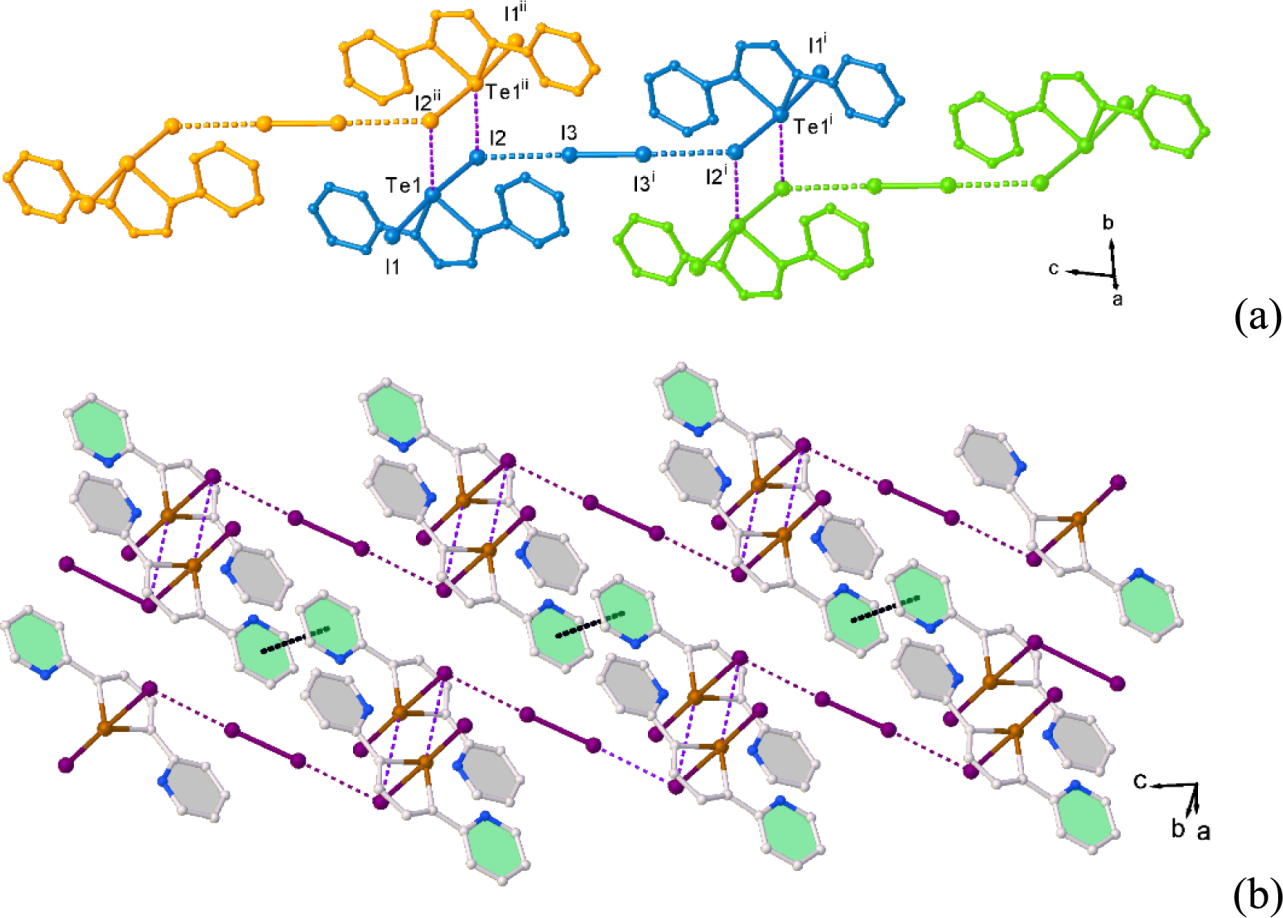
Partial views of the crystal packing in **L**I_2_·1/2I_2_ (**2**) showing the Te···I interactions between “Z-shaped” **L**I_2_···I_2_···**L**I_2_ motifs (a); and π−π stacking interactions between the infinite ···{**L**I_2_···I_2_···**L**I_2_}_∞_··· chains (b). Symmetry codes: ^i^ = 1−*x*, 2−*y*, − *z*; ^ii^ = 1−*x*, 2−*y*, 1− *z*.

Following our aim to comparatively analyze the structural features determined by σ-hole interactions such as HaBs and ChBs, within a family of related compounds, we reacted **L** with Br_2_ in 1:1 and 1:2 reaction molar ratios. Orange lath-shaped crystals were obtained in both cases from a 2:1 (v/v) mixture of CH_3_CN/CH_2_Cl_2_, corresponding to the formulation **L**Br_2_ (**3**). X-ray diffraction analysis allowed us to establish the oxidative addition of the dihalogen to the tellurium­(II) atom of **L** and the formation of a hypercoordinate tellurium­(IV) adduct featuring a seesaw geometry at the chalcogen center similar to that observed for **1** and **2** [Te1−Br1 = 2.6482(3), Te1−Br2 = 2.6690(3) Å; Br1−Te1−Br2 = 172.585(8)°] (Tables S1, S5 and Figure 5).

**Figure 5:**
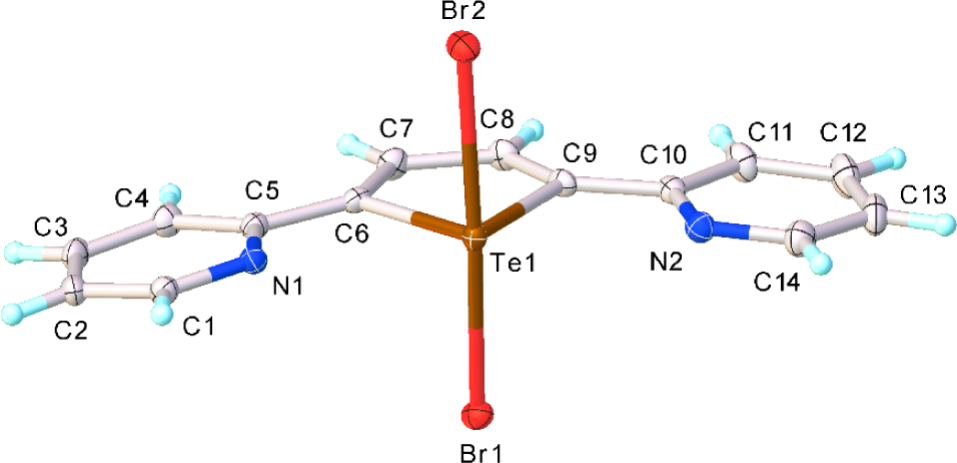
X-ray crystal structure of **L**Br_2_ (**3**) showing the atom labeling scheme adopted. Thermal ellipsoids are drawn at 50% probability level.

In contrast to what observed for **1** and **2**, in **3** the crystal packing is mainly determined only by halogen···halogen interactions between adjacent adduct units giving rise to infinite zigzag chains of halogen-bonded **L**Br_2_ units, which develop parallel to the [101] direction (Br1···Br2^i^ = 3.6196 (4) Å; Figure 6).

**Figure 6:**
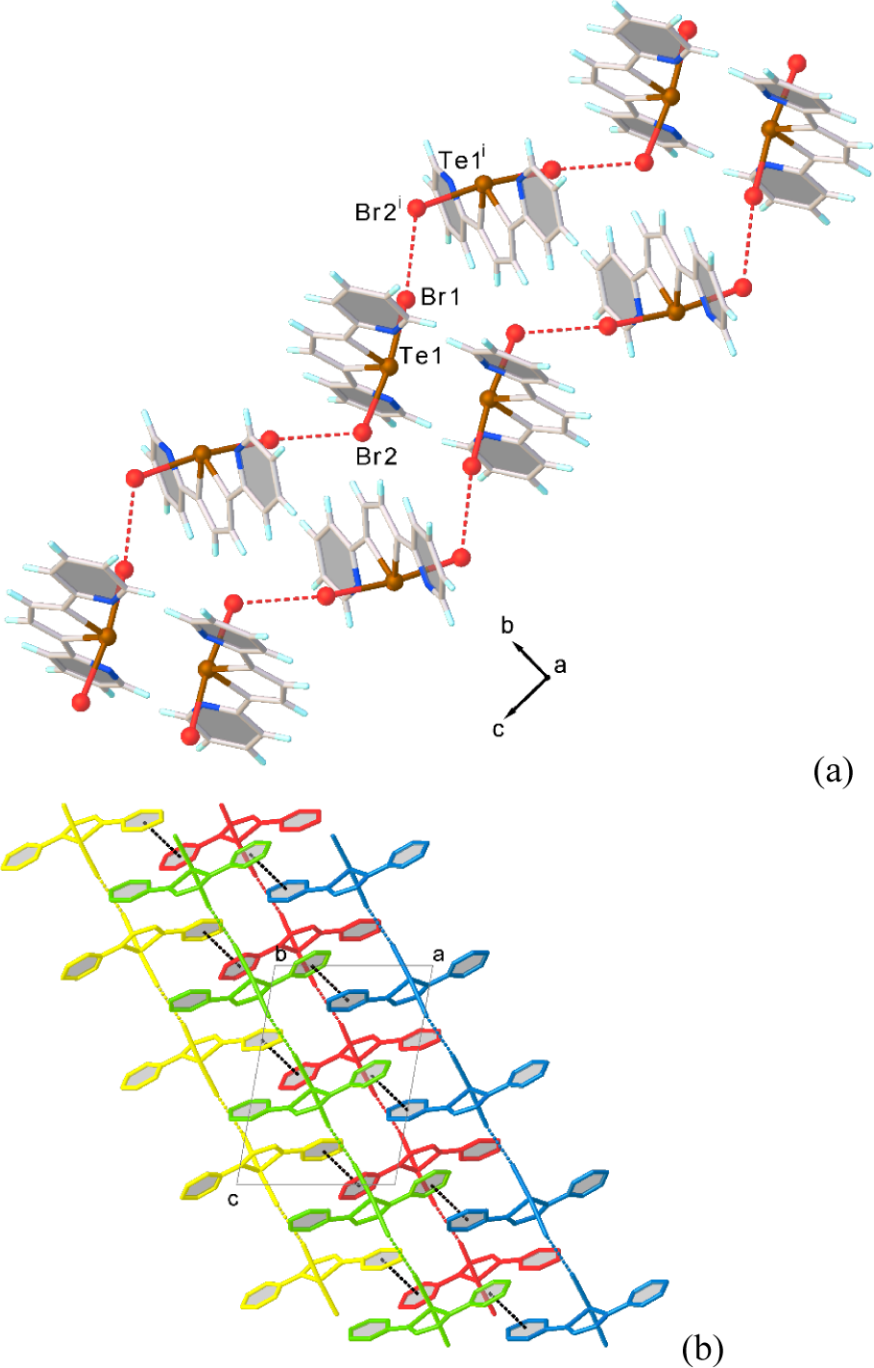
Partial view along the *a*-axis of infinite chains of halogen-bonded adduct units in **L**Br_2_ (a); partial view along the *b*-axis of the packing diagram in **L**Br_2_ (b). Symmetry code: ^i^ = − 1/2+*x*, 3/2−*y*, − 1/2+*z*.

Considering the Te−Br···Br−Te moieties within the chains, the significantly unequal values of the Te−Br−Br angles of 172.52(2)° (Te1−Br1···Br2^i^ in Figure 6a) and 122.34(5)° (Te1^i^−Br2^i^···Br1 in Figure 6a), and their difference of about 50°, are consistent with the formation of type II HaBs between the **L**Br_2_ units.^88^ The σ-hole on the Br atom of one unit interacts with the negative belt on the Br atom of an adjacent unit so that each adduct behaves as HaB acceptor at one bromine side and HaB donor at the other bromine side. Slipped π−π stacking interactions (intercentroid distance = 3.92 Å, shift distance = 1.19 Å) between pyridyl rings of **L**Br_2_ units from parallel infinite chains determine the crystal packing (Figure 6b).

The reactivity of **L** toward IBr and ICl was also investigated under the same experimental conditions used for I_2_ and Br_2_ (see Experimental section). Red rod-shaped crystals (compound **4** in the case of IBr) and orange block-shaped crystals (compound **5** in the case of ICl) were obtained with good reproducibility on average from a 2:1 (v/v) mixture of CH_3_CN/CH_2_Cl_2_, which gave elemental analysis results different from those expected for 1:1 **L**IBr and **L**ICl formulations, respectively. X-ray diffraction analysis confirmed in both cases the oxidative addition of a dihalogen molecule to the tellurium­(II) atom of **L**. For both **4** and **5**, during structure refinement using the seesaw Br−Te­(**L**)−Br and Cl−Te­(**L**)−Cl models, respectively, anomalous displacement parameters for both halogen sites, in both compounds, suggested that these sites might be partially occupied by iodine. Taking this into account, the refinement of the disordered models gave the best fit with Br/I occupancies of 0.85/0.15 and 0.78/0.22 for the two halogen sites in **4**, and Cl/I occupancies of 0.91/0.09 and 0.95/0.05 for the two halogen sites in **5**. Therefore, insertion adducts **4** and **5** should more accurately be formulated as **L**Br_1.63_I_0.37_ and **L**Cl_1.86_I_0.14_, respectively, in agreement with microanalytical data from the crystal crops analyzed (Tables S1, S6, and S7 and Figure S4). In both compounds, the X−Te−X moieties [X = Br/I (**4**) and Cl/I­(**5**)] are almost linear, and roughly perpendicular to the tellurophene penta-atomic ring as observed in compounds **1**−**3**. Since the measured electron density corresponding to the X−Te−X moieties [X = Br/I (**4**) and Cl/I­(**5**)] is an average of the contents of all unit cells in the crystals, the fitting of the data adopting a disordered model is unable to give a clear indication of the presence in the crystals of discrete molecular adducts containing the I−Te−Br, I−Te−I, or Br−Te−Br moieties in the case of **4**, and the I−Te−Cl, I−Te−I, or Cl−Te−Cl moieties in the case of **5** (i.e., the presence of I−Te−I moieties cannot be excluded in both cases). These results are consistent with the tendency of interhalogens IX to disproportionate giving rise to I_2_ and X_2_ (X = Br, Cl) molecules,^53,54,95^ which in turn can undergo oxidative addition to the tellurophene molecule. Interestingly, both compounds **4** and **5** are isostructural with **3**, and feature type II HaBs between the halide components of 3.6047(5) Å in the case of **4** and 3.7079(9) Å in the case of **5**.

It is quite interesting to compare it with the structural features of analogous hypercoordinate Te­(IV) compounds obtained from the π-conjugated tellurophenes **L** ^**a**^−**L** ^**f**^. In compounds **L** ^**a,b,d,e**^Br_2_ no Te···Br intermolecular interactions are observed between the corresponding asymmetric units (interacting via C−H···Br contacts) featuring a similar seesaw geometry at the chalcogen center. This could be attributed to the steric hindrance caused by the substituents on the phenyl rings. On the other hand, in the case of **L** ^**c**^Br_2_, the two −C_6_F_5_ per-haloaryl groups in the adduct unit do not lie on the same plane of the tellurophene ring as observed in **1**−**5**, but they define a dihedral angle of approximately 43°. This allows each adduct unit to form Te···Br ChBs with two neighboring units, thus generating 1D ribbons in the crystal packing of Te_2_Br_2_ *pseudo*-square motifs sharing two vertices (Figure S5). In the isostructural **L** ^**f**^Br_2_ and **L** ^**f**^Cl_2_, the phenyl rings are also not coplanar with the tellurophene core, exhibiting twisted dihedral angles of up to 33°. However, in these compounds, only one halogen site of the X−Te­(**L** ^**f**^)−X fragment is engaged in a Te···X ChB (X = Br, Cl) with a neighboring unit, thus forming zigzag −Te···X−Te···X−Te··· chains in the crystal packing (Figure S6). Therefore, it can be inferred that the coplanar conformation assumed by **L** and the absence of substituents on the pyridyl moieties could be responsible for the peculiar Te···X (X = I, Br) intermolecular interactions and crystal packing observed in **1**−**5**.

Intrigued by the structural comparison presented above, we have performed a CCDC search for compounds featuring a central chalcogen atom (S, Se, and Te) bound to two halogen atoms (X) and to two other atoms (E) of elements different from halogens with a coordination number (CN) of 4 (Figure S7). Moreover, as many as 467 out of 588 hits feature a linear X−Ch−Y fragment (X = Y = I, Br, Cl, F; X = I, Y = Cl, Br, F) and a dihedral angle of about 90° between the plane containing the X−Ch−Y fragment and that containing the E−Ch−E one (Figure S8). 416 of these structures feature at least one C atom bound to the central chalcogen atom in a seesaw geometry. Interestingly, no compounds featuring I−Ch−Y (Ch = S, Se) and Br−S−Y (Y = I, Br, Cl, F) moieties are known with the chalcogen atom having a CN = 4 (Figure S9).

### FT-Raman Characterization

FT-Raman spectroscopy has been largely exploited to investigate the structure of compounds formed from the reactions of chalcogen donors with elemental dihalogens,^37,67,80,96,97^ especially in those cases where the X-ray crystal structure is not available.

The FT-Raman spectrum (60−500 cm^−1^) of **1** shows a strong band centered at 110 cm^−1^, which can be assigned to the symmetric stretching vibration of the I−Te−I moiety. This assignment can be considered as reasonable since the almost symmetric I−Te−I three-body system in **1** can be considered structurally comparable to a symmetric/slightly asymmetric I_3_^−^ (I−I bond lengths in the range 2.90−3.00 Å), which is characterized by an intense Raman band centered at about 110 cm^−1^ due to the σ_g_ symmetric stretching vibration of the I−I−I three-body system. Accordingly, a Raman band positioned at a very close wavenumber (115 cm^−1^) was observed in the case of the “T-shaped” compound H^o^PyTeI_2_ (H^o^Py = N-protonated 4-pyridyl).^80,98^

The FT-Raman spectrum of compound **2** is more structured compared to that of **1** and presents three bands in the low frequency region centered at 110, 143, and 176 cm^−1^. Considering that in **2**, the I−Te−I moiety is more asymmetric than in **1**, the first two bands can be assigned to its symmetric and antisymmetric stretching vibrations, respectively, identified at 115 and 156 cm^−1^, respectively, in the case of H^o^PyTeI_2_.^80,98^ This is in agreement with the FT-Raman spectrum typically recorded for an asymmetric I_3_^−^ (I−I bond lengths in the range 2.80−3.10 Å), which beside the band at about 110 cm^−1^, also presents a band centered at about 140 cm^−1^ due to the σ_u_ antisymmetric stretching vibration of the I−I−I three-body system [calculated normal-mode frequencies for I_3_^−^ (*D*_∞h_) in the gas phase are 58 (π_u_), 109 (s, σ_g_), and 134 (σ_u_) cm^−1^]. The band centered at 176 cm^−1^ can be assigned to the stretching vibration of the cocrystallized I_2_ molecule bridging two **L**I_2_ TS-adduct units in **2**. In fact, solid-state I_2_ (I−I bond length = 2.715 Å) shows a peculiar FT-Raman band centered at 180 cm^−1^, due to the I−I stretching vibration, that linearly shifts toward lower wavenumbers on lengthening the I−I bond.^37,67,80,96,97^

The FT-Raman spectrum of compound **3** is dominated by a strong band at 159 cm^−1^ accompanied by two less intense ones centered at 98 and 171 cm^−1^. These bands fall at the same energy as those recorded in the case of H^o^PyTeBr_4_ (158, 168, and a broad band in the range 80−100 cm^−1^, respectively).^80^ As already shown for the vibrational properties of Br−Se−Br three-body systems that resemble those of the [Br−X−Br]^−^ (X = I, Br) anions, the bands observed in the FT-Raman spectrum of **3** can also be interpreted in terms of these structural and atomic mass similarities of the oscillators responsible for the observed FT-Raman bands. In fact, the strong band centered at 159 cm^−1^ can be assigned to the symmetric (σ_g_) stretching vibration of the Br−Te−Br three-body system in **3**, while the those at 171 and 98 cm^−1^ could be assigned to the antisymmetric (σ_u_) stretching and bending (π_u_) vibrations, respectively [calculated normal-mode frequencies for XBr_2_^−^ (X = I, Br) anions in the gas phase are 89 (π_u_), 156 (s, σ_g_), and 181 (σ_u_) cm^−1^ for Br_3_^−^ (D_∞h_), and 76 (π_u_), 153 (s, σ_g_), and 163 (σ_u_) cm^−1^ for IBr_2_^−^ (D_∞h_)].^97^

The observed complexity of the FT-Raman spectra of compounds **4** and **5** in the low frequency region reflects the disorder in their crystal structures and supports the simultaneous presence of different hypercoordinate tellurium­(IV) systems in the solid state. The FT-Raman spectrum of **4** is characterized by three main bands centered at 112 (with a shoulder at 105 cm^-1^), 134, and 159 cm^−1^ (with a shoulder at 166 cm^−1^). Following the discussion above, the first and third bands could be considered as mainly contributed by the symmetric stretching vibrations of I−Te−I and Br−Te−Br moieties, respectively. The band positioned at 134 cm^−1^ could see contributions from both the σ_u_ vibration mode of an I−Te−I fragment (see above) and from an I−Te−Br moiety, which is similar in mass to the anion I_2_Br^−^ [calculated normal-mode frequencies for I_2_Br^−^ anion in the gas phase are 69 (π_u_), 112 (s, σ_g_), and 162 (σ_u_) cm^−1^ (D_∞h_), and 67 (π_u_), 124 (s, σ_g_), and 155 (s, σ_u_) cm^−1^ (C_∞h_)]. The vibrational modes of the I−Te−Br moiety could also contribute to the bands observed for **4** at 112 and 159 cm^−1^.^97^

The FT-Raman spectrum of compound **5** shows a broad and intense band centered at 105 cm^−1^ and less intense bands at 148, 213, and 266 cm^−1^. The latter two can be considered to be mainly contributed by the antisymmetric (σ_u_) and symmetric (σ_g_) stretching vibrations of the Cl−Te−Cl fragment, respectively, in agreement with the corresponding values calculated at the hybrid-DFT level for the anion ICl_2_^−^ (D_∞h_, σ_u_ = 231, σ_g_ = 247 cm^−1^).^97^ The π_u_ bending mode of the Cl−Te−Cl fragment (calculated at 108 cm^−1^ for ICl_2_^−^) could contribute to the band observed for **5** at 105 cm^−1^ together with the symmetric stretching of the I−Te−I fragment. A contribution to the FT-Raman bands observed for **5** at 148 and 221 cm^−1^ can also be expected from the vibrational modes of the I−Te−Cl moiety; in fact the two Raman-active stretching modes for the similar anionic three-body system I_2_Cl^−^ are calculated at 133 and 221 cm^−1^ in the gas phase, the former featuring a major contribution from the ν­(I−I) stretching vibration and the latter from the ν­(I−Cl) one.^99^

### Theoretical Calculations

In principle, the reactions of **L** with elemental dihalogens (XY, X = Y = I, Br, Cl; X = I, Y = Cl, Br) could provide several types of products, including CT-adducts containing the N−X−Y moiety, CT-adducts containing the Te−X−Y moiety, and hypercoordinate adducts featuring a X−Te−Y seesaw motif. In addition, pyridyl donors could can also undergo N-protonation to give cations counterbalanced by discrete or extended (poly)­halides.^80,97,99^

In order to investigate the reasons responsible for the formation of the isolated reaction products, the electronic structure of **L** was initially evaluated at the density functional theory (DFT) level^100^ in the gas phase (mPW1PW functional; Def2-SVP basis set for light atomic species; LANL08­(d) for Te and halogens; see Experimental Section for details). The most stable geometry was found to show the pyridine rings lying on the plane of the tellurophene ring in a synperiplanar conformation, in accordance with the geometrical features of compounds 1−**5** discussed above. This conformation is indeed stabilized by a weak interaction (2.93 kcal/mol) involving the lone pairs of electrons (LPs) of both nitrogen atoms of the pyridine rings and the antibonding natural orbital localized on the C−Te bonds of the tellurophene ring. The possibility of **L** to behave as a donor toward dihalogens can be hypothesized considering the presence of LPs on both N and Te atoms; this is indeed supported by their localization in the calculated Kohn−Sham occupied frontier molecular orbitals (KS-MOs; Figure 7).

**Figure 7:**
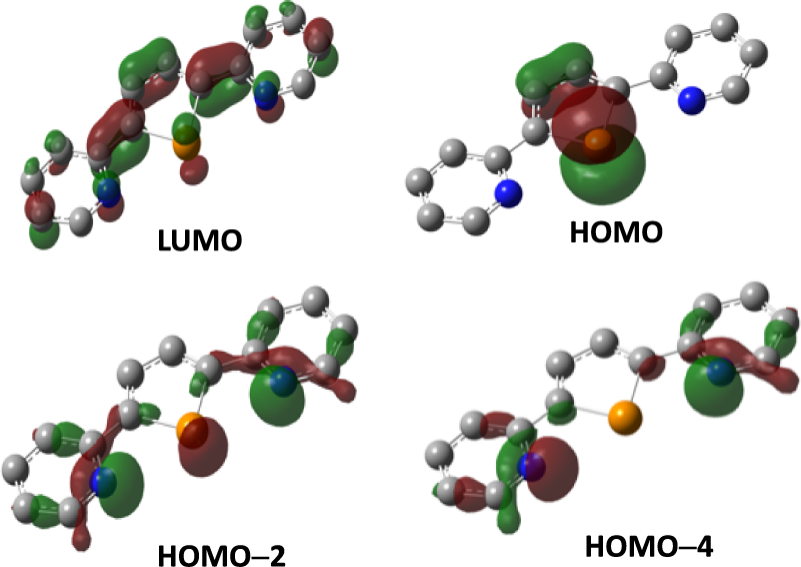
Isosurfaces of selected Kohn−Sham molecular orbitals calculated for L. Color code: carbon, gray; nitrogen, blue; tellurium, orange. Hydrogen atoms are omitted for clarity. Cutoff value: 0.05 |e|.

DFT calculations were extended to the possible neutral reaction products to verify their relative stability. As regards 1:1 CT-adducts, the KS-MO composition calculated for **L** is consistent with the possible formation of CT-adducts either at the N-donors with the dihalogen molecule lying in the plane of the pyridine ring or at the Te-donor with the dihalogen molecule perpendicular to the pentatomic telluracycle. While the LP eigenvalue at the optimized geometry on the Te-atom (HOMO) assumes less negative eigenvalues than those on N atoms (HOMO-2 and HOMO-4), the charge distribution calculated at NBO level^69^ suggests a larger negative charge on the pyridine nitrogen atoms as compared to tellurium atom (*Q*_N_ = −0.508 |e|; *Q*_Te_ = +0.920 |e|).

With the aim of evaluating the different stabilities of the potential reaction products, both 1:1 CT “spoke” adducts at the N/Te-donor atoms and 1:1 seesaw Te-adducts were optimized (see Figure 8 for the case of **L**/I_2_).

**Figure 8:**
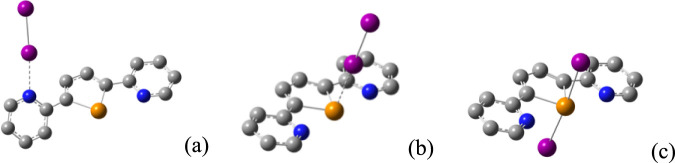
Representations of the 1:1 CT-adduct at the N-donor atom (a), Te-donor atom (b), and seesaw adduct (c) optimized for the case L/I_2_ at the DFT level. Color code: carbon, gray; nitrogen, blue; tellurium, orange; iodine, purple. Hydrogen atoms are omitted for clarity.

As far as the seesaw complexes are concerned, a very good agreement was found between the optimized bond lengths and angles and the corresponding structural data refined for compounds **1**−**5** (see above). In particular, the calculated geometric features of the X−Te−Y (X = Y = I, Br, or Cl; X = I, Y = Cl, or Br) moieties, summarized in Table 1, satisfactorily reproduce the experimental data.

**Table 1:** Optimized Te−X/Y Bond Lengths [*d*_Te-X/Y_ (Å)], Natural Charges *Q* on the Te and X/Y Atoms (|e|), Wiberg Bond Indices (WBI), and Raman-Active Vibrational Stretching Frequencies [ν (cm^−1^)] Calculated at DFT Level for the Seesaw Adducts LXY (X = Y = I, Br, Cl; X = I, Y = Cl, Br)

	*d*_Te−X_ ^a^	*d*_Te−Y_ ^a^	*Q*_Te_	*Q*_X_	*Q*_Y_	WBI_Te−X_	WBI_Te−Y_	ν_Te−X_	ν_Te−Y_
**L**	−	−	0.920	−	−	−		−	
**L**Cl_2_	2.520	2.520	1.626	−0.526	−0.526	0.537	0.537	266.1	
**L**Br_2_	2.702	2.702	1.485	−0.457	−0.457	0.548	0.548	162.8	
**L**I_2_	2.927	2.927	1.345	−0.386	−0.386	0.538	0.538	115.3	
**L**ICl	2.541	2.902	1.485	−0.527	−0.381	0.490	0.602	262.0	146.1
**L**IBr	2.713	2.915	1.415	−0.458	−0.384	0.520	0.570	168.6	133.6

a When X≠Y, X is the lightest halogen species.

Worthy of note, in agreement with the structural data described above for compounds **1**−**5**, a weak N···Te interaction, described above for the free ligand **L** and involving both N atoms, was found to stabilize the periplanar conformation between the pyridine and the tellurophene rings in hypercoordinate adducts **L**XY (2.07, 3.65, 3.93, 2.23, and 3.66 kcal/mol for XY = Cl_2_, Br_2_, I_2_, IBr, and ICl, respectively). The nature of the MOs calculated for the seesaw adducts is in agreement with the Rundle−Pimentel (RP) description^101,102^ of the X−Te−X/Y three-body systems.

In Figure 9, the occupied bonding (KS-MO #52), occupied nonbonding (KS-MO #64, KS-HOMO), and unoccupied antibonding MOs (KS-MO #66, KS-LUMO+1) are represented for **L**I_2_. Worthy of note, in agreement with the RP model, the sum of Wiberg bond indices^103^ for Te−X/Y bonds in **L**XY seesaw adducts is close to unity.

**Figure 9:**
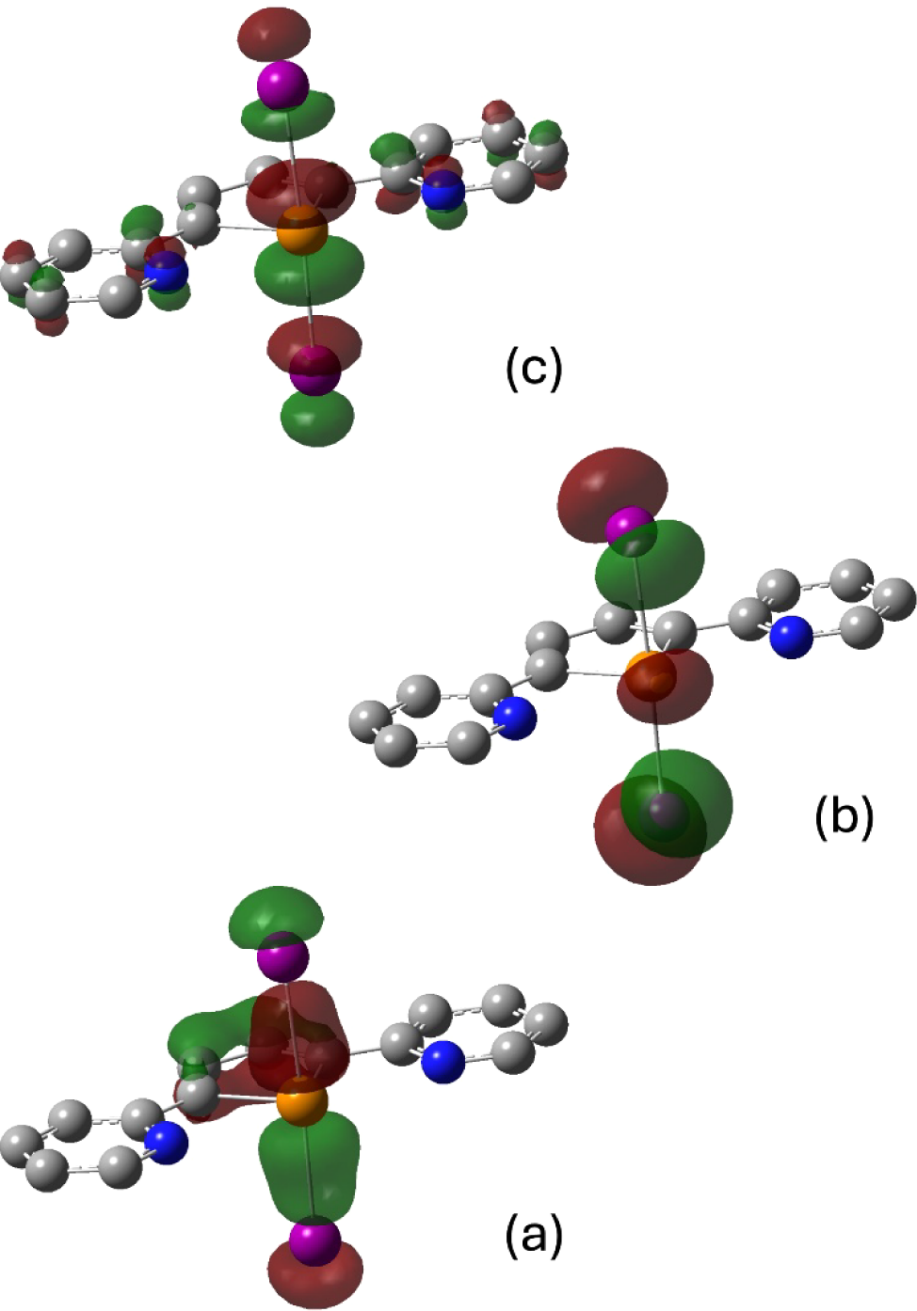
Bonding (a), nonbonding (b), and antibonding (c) KS-MOs that concur to the bond of the three-body system I−Te−I in LI_2_ at the DFT-optimized geometry. Color code: carbon, gray; nitrogen, blue; tellurium, orange; iodine, purple. Hydrogen atoms are omitted for clarity. Cutoff value = 0.05 |e|.

As far as CT-adducts are concerned, those at the N-donors feature the dihalogen molecule lying in the plane of the pyridine ring, as expected; furthermore, the pyridine ring involved in the donor−acceptor interaction is rotated with respect to the plane containing the pentatomic telluracycle. In the case of **L**·Cl_2_ and **L**·Br_2_ CT-adducts, two stable energy minima were optimized, featuring the N···X−Y moiety oriented in a *pseudo*-periplanar or *pseudo*-antiperiplanar (Figure 8a for of **L**·I_2_) conformation with respect to the tellurophene ring. The most stable conformers show the pyridine ring rotated by a dihedral angle (Te**−**C**−**C**−**N) dependent on the steric hindrance of the coordinated halogen [τ = 18.05, 28.07, 144.49, 139.78, and 141.97° for **L**·Cl_2_, **L**·Br_2_, **L**·I_2_, **L**·ICl, and **L**·IBr, respectively; Figure 8a for **L**·I_2_ (the angles closer to zero (or generally <90°) define *pseudo*-periplanar orientations, while the others, closer to 180° (or generally >90°), define *pseudo*-antiperiplanar orientations)]. The entity of the CT is calculated to be modest (0.141−0.176 |e|), and the elongation of the coordinated dihalogen does not exceed 4.4% in **L**·Cl_2_ (Table S8).

The CT-adducts at the tellurium atom show the dihalogen molecule perpendicular to the plane of the ligand and are calculated to be remarkably stronger than the corresponding adducts at the N atom. The entity of the CT calculated at NBO level decreases on passing from X = Cl to X = I (Table S8) in **L**·X_2_ CT-adducts (0.394 and 0.274 |e|, respectively) and is determined by the nature of the terminal Y atom in **L**·XY adducts (X = I; |CT| = 0.335 and 0.307 |e| for Y = Cl and Br, respectively). The amount of calculated CT is reflected in the elongation of the halogen−halogen bond in the optimized adduct as compared to the free XY molecule (X = Y = I, Br, Cl; X = I, Y = Cl, Br; relative elongation = 10.3, 6.4, 4.4, 6.0, and 5.2% for Cl_2_, Br_2_, I_2_, ICl, and IBr, respectively) and in Wiberg bond indices following the same trend (Table S8). Consequently, the CT is linearly correlated to the relative elongation of the coordinated dihalogen molecule (Figure S10).

The charge separation within the coordinated dihalogen moiety is only partially dependent on the CT, being strongly influenced by the electronegativity difference between the two halogen atoms (Table S8). Not surprisingly, the largest polarization is achieved in the **L**·ICl CT-adduct. Therefore, at least within this class of CT-adducts, the entity of CT and the corresponding elongation of the interhalogen bond length should not be considered as an index of the polarization of the bond.

Thermochemical analysis was carried out to evaluate the relative stabilities of the possible CT and seesaw adducts (Table S9). The formation of 1:1 CT-adducts was generally calculated to be endoergonic (Δ*G*_f_ > 0 for the Cl_2_, Br_2_, I_2_, and IBr CT-adducts at the N-donor, and for the I_2_ CT-adduct at the Te-donor atom) or only slightly exoergonic (Δ*G*_f_ < 0 for the ICl CT-adduct at the N-donor, and for the Br_2_, ICl, and IBr CT-adducts at the Te-donor atom). On the contrary, the formation of seesaw adducts at the Te-donor atom is always favored (Δ*G*_f_ ranging between −30.5 and −1.8 kcal/mol). As a result, the formation of seesaw adducts at the tellurium center is largely favored as compared to CT-adducts at the N-donor (between 5.6 and 35.2 kcal/mol for **L**I_2_ and **L**Cl_2_, respectively) and CT-adducts at the Te-donor atom (between 2.1 and 31.6 kcal/mol for **L**I_2_ and **L**Cl_2_, respectively). Notably, the adduct formation enthalpy values Δ*H*_f_ indicate that the formation of both 1:1 CT and seesaw adducts, although to a very different extent, is always exothermic, so that entropy variations are relevant in the formation of the reaction products. Hence, the thermochemical analysis accounts for the isolation of seesaw adducts at the tellurium­(II) center in place of CT-adducts.

As previously mentioned, the preferential outcome of the reactions between organochalcogen­(II) donors (RCh, R_2_Ch; R = organic framework; Ch = S, Se, Te) with elemental dihalogens (XY, X = Y = I, Br, Cl; X = I, Y = Cl, Br) can be predicted by hypothesizing as intermediate the [RCh−X]^+^ cation. To the best of our knowledge, the cation intermediate was first reported by Detty et al. in 1994:^71^ the oxidative addition of Br_2_ to organoselenium­(II) and organotellurium­(II) compounds could be interpreted by hypothesizing an initial fast η^1^-association of bromine to form a CT-adduct RCh·Br_2_, that could evolve to an ionic couple [RCh−Br]^+^Br^−^, in equilibrium with the hypercoordinate species. A few years later, Husebye et al. proposed a similar ionic intermediate in the reactions of trisubstituted phosphaneselenide with elemental diiodine.^70^ More recently, Carrera et al. investigated the bromination of substituted tellurophene derivatives **L** ^**a−d**^ (Scheme 1) to form seesaw addition products and proposed a similar dissociative reaction path involving first an η^1^-association complex (CT-adduct) followed by the formation of a ionic [**L** ^**a−d**^−Br]^+^ intermediate. An alternative mechanism was also proposed, involving first an η^2^-association complex that could directly origin the final hypercoordinate product without the formation of a [**L** ^**a−d**^−Br]^+^ intermediate.^55^ In this context, it is important to recall that in the case of the reactions of 1,3-dimethylimidazoline-2-thione and -2-selone with elemental dihalogens, the η^2^-species was calculated to be a transition state in the fluxional interconversion between CT and hypercoordinate adducts.^73^ We showed that the natural charge distribution on such hypothetical [RCh−X]^+^ cations (RCh = imidazole- and thiazole-2-chalcogenone derivatives; X = I, Br), which show a σ*-LUMO localized along the Ch−X bond, could systematically predict the nature of the final products, including CT “spoke” and hypercoordinate “T-shaped” adducts as well as [(RCh)_2_]^2+^ dications featuring a Ch−Ch bond.^72^

Therefore, the geometry of [**L**X]^+^ cations (X = Cl, Br, I), featuring a Te−X bond, was optimized, and their natural charge distribution was evaluated. The KS-LUMO of the [**L**X]^+^ cations, as expected, is aligned along the Te−X bond, thus allowing the hypothetical cation intermediate to receive electron density on either the Te atom (to give seesaw adducts) or the X atom (to give CT-adducts). Considering that in all cases the Te atom was calculated to display a positive charge much higher than that on the X atom, the formation of the seesaw adduct can be foreseen to be favored with respect to the CT-adduct, in perfect agreement with the calculated thermochemical data and experimental data.

Although [RCh−X]^+^ cation intermediates should be considered only as a useful tool to predict the most likely products from the reactions of chalcogen donors with elemental dihalogens, it is conceivable that the formation of CT-adducts is the first step occurring in solution.^55,70–73^ The charge-transfer interaction between the reactants results in the polarization of the XY coordinated dihalogen molecule within the [RCh−X^δ+^···Y^δ−^] species (X = Y = I, Br, Cl; X = I, Y = Cl, Br). This species could eventually evolve to the final seesaw adduct via the [RCh−X]^+^ cation depending on the nature of the chalcogen donor, dihalogen acceptor, and the solvent.^72^

The calculated natural charge distribution within **L**·XY CT-adducts at the Te-donor atom shows that a CT as large as 0.393, 0.331, 0.274, 0.335, and 0.307 |e|, for Cl_2_, Br_2_, I_2_, ICl, and IBr, respectively, occurs from **L** to the coordinated dihalogen molecule. Notably, this corresponds to a different degree of polarization of the coordinated dihalogen (Δ*Q*_X-Y_ = 0.078, 0.098, 0.115, 0.488, and 0.314 |e| for XY = Cl_2_, Br_2_, I_2_, ICl, and IBr, respectively), so that the CT-adducts with dihalogens featuring X≠Y are those giving the largest charge separation.

We have also calculated at DFT level the Raman-active vibrational stretching frequencies [ν (cm^−1^)] for the seesaw adducts **L**XY (X = Y = I, Br, Cl; X = I, Y = Cl, Br, see Table 1). In the cases of **L**Cl_2_, **L**Br_2_, and **L**I_2_, the most intense calculated mode (266.1, 162.8, and 115.3 cm^−1^, respectively) is the low-energy symmetrical stretching vibration of the X−Te−X moiety coupled with an in-plane in-phase bending of the pyridine substituents. These calculated frequencies are in good agreement with the experimental ones recorded for **1**−**5** and with the assignments made based on the similarities with the vibrational properties of the anions I_3_^−^ and XBr_2_^−^ (X = I, Br, Cl) (see above).^37,80,97^

In the case of the seesaw adducts **L**IBr and **L**ICl, two main vibrational modes are calculated to be Raman-active, which involve the independent stretching vibrations of the Te−I and the Te−Y (Y = Br, Cl) bonds combined with both the torsion of the pyridine rings with respect to the central pentatomic telluracycle and with the libration of this latter ring with respect to the X−Te−Y moiety (**L**IBr: 133.6 and 168.6 cm^−1^; **L**ICl: 146.1 and 262.0 cm^−1^, see Table 1). In these cases as well, the calculated Raman frequencies agree satisfactorily with the experimental bands observed in the Raman spectra recorded for **4** and **5**, and with the assignments made based on the vibrational similarities to the anions I_2_X^−^ (X = Br, Cl, see above).

### Supramolecular Interactions

Structural analysis of the compounds obtained from the reactions of **L** with elemental dihalogens and characterized by single-crystal X-ray diffraction revealed that their crystal packing is governed by a combination of HB, HaB, and ChB interactions. HaB and ChB are defined as net attractive interactions R−X/Ch···A between an HaB/ChB donor (R = heteroatom, metal ion, organic group, X = halogen, Ch = chalcogen) and a bond acceptor A.^1–15,104–107^

HaB and ChB are usually classified within the σ-hole family of intermolecular interactions, while rarer π-hole HaB^108^ and π-hole ChB^109^ interactions are also possible. These NCIs are characterized by a region of depleted electrostatic potential (σ-hole) that forms on the surface of the X/Ch atom (typically disposed opposite to the covalent R−X/Ch bond of the donor group), exhibiting electrophilic properties due to its positive—or less negative—electrostatic potential. These σ-hole interactions are determined by the anisotropic distribution of the electrostatic potential around the interacting atoms. Energy decomposition analyses (EDAs) have shown that σ-hole interactions can be dependent both on an electrostatic term and an MO-mixing term,^110,111^ in addition to dispersion effects.^112^ The relative contribution of each term is largely dependent on the nature of the interacting groups involved. Consequently, the nature of σ-hole interactions can span from almost purely ionic to largely covalent.

A map of the electrostatic potential can highlight electrophilic (positive − or less negative − electrostatic potential) regions on the bond donor R−X/Ch and nucleophilic regions (negative − or less positive − electrostatic potential) on the bond acceptor A. Such electrostatic analysis can be combined with the study of antibonding (BD*) NBOs on the R-X/Ch bond and LPs on the A group, thus allowing a DFT investigation of the electrostatic and MO-mixing nature of ChB and HaB interactions.

The mapped electrostatic potentials of the TS-adducts **L**Cl_2_, **L**Br_2_, and **L**I_2_ are depicted in Figure 10.

**Figure 10:**
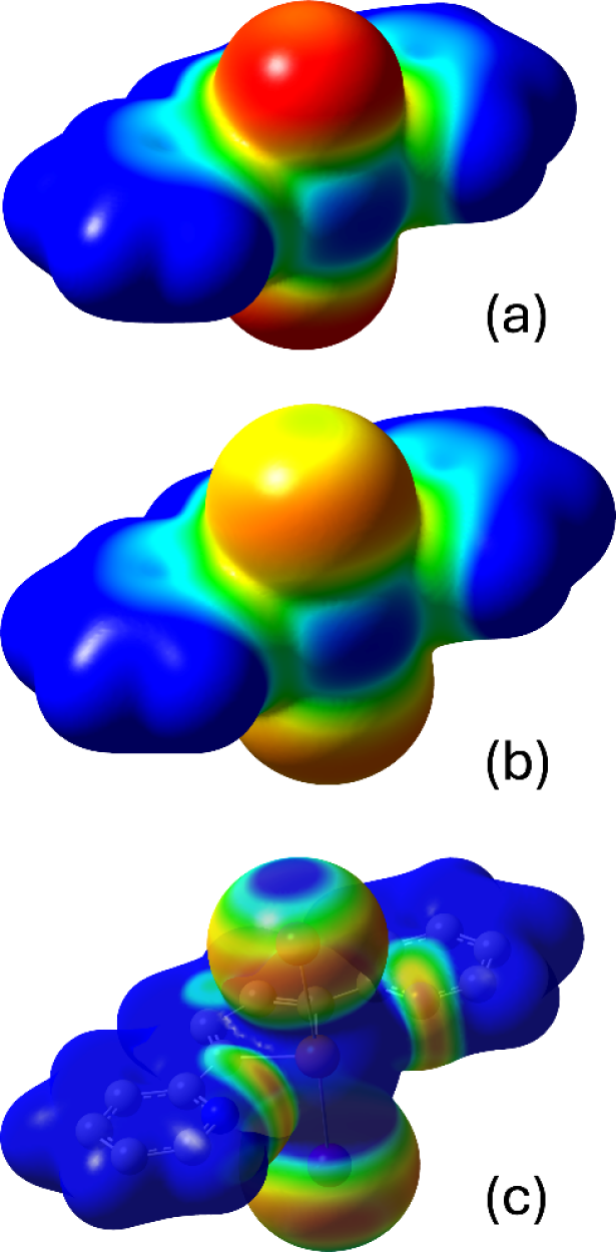
Molecular electrostatic potential mapped on the electron density [5·10^−3^ |e|/Bohr^3^; range −0.035 (red) to +0.000 (blue) a.u.] calculated for the seesaw adducts **L**Cl_2_ (a), **L**Br_2_ (b), and **L**I_2_ (c) at the DFT level. Hydrogen atoms are omitted for clarity.

In all cases, the halogen atoms display a negative electrostatic potential, unevenly distributed, thus displaying a σ-hole opposite the Te−X bond (X = Cl, Br, I) and a belt of negative charge surrounding the X atom perpendicularly to the same bond. An analysis of the electrostatic potentials at the halogens clearly points to **L**I_2_ as that showing the most pronounced anisotropy (Cl: −0.041, −0.045; Br: −0.030, −0.040; I: +0.007, −0.040 au; Figure 11). Such σ-hole topology closely resembles that discussed previously for several compounds featuring chalcogen-halogen or halogen−halogen bonds.^63,80,107^ In addition, a further charge depletion of +0.042 au can be spotted on the positively charged Te atom (Figure 11), perpendicular to the I−Te−I moiety.

**Figure 11:**
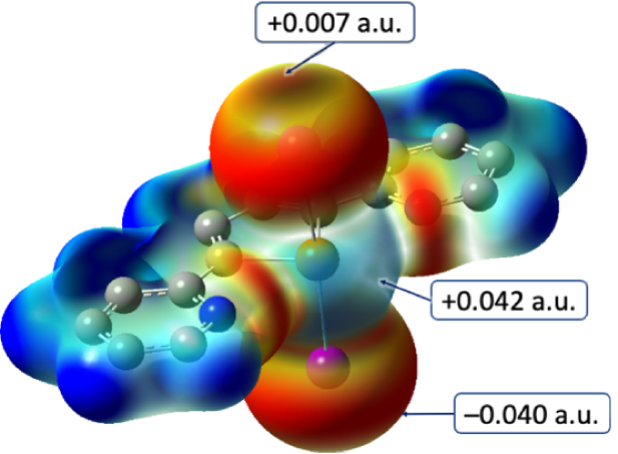
Molecular electrostatic potential (MEP) mapped on the electron density [5·10^−3^ |e|/Bohr^3^; range −0.040 (red) to +0.007 (blue) a.u.] calculated for compound **L**I_2_ at the DFT level. Hydrogen atoms are omitted for clarity.

This σ-hole justifies both type-I and type-II interactions and Te−I···Te HaB interactions observed in the crystal structure of compounds **1** and **2** (see above). This σ-hole at the Te atoms is much less pronounced in the cases of the TS-adducts **L**Cl_2_, **L**Br_2_; in fact, no Te−X···Te (X = Br, Cl) HaB interactions are observed in the structures of compounds **3**−**5**.

Therefore, the computed electrostatic maps agree with the geometric features of Te1−I1···I2^ii^ and Te1···I2^i^−Te1^i^ interactions in compound **1** (Figure 2), Te1−I2···I3 and Te1···I2^ii^-Te1^ii^ in compound **2** (Figure 3), and Te1−Br1···Br2^i^ in compound **3** (Figure 6), thus nicely accounting for the structural features of the crystal packing described above.

## Experimental Section

### Materials and Instruments

Reagents and solvents of reagent grade purity were used as received from Aldrich. Compound **L** was prepared as described previously^87^ and recrystallized from CHCl_3_ as yellow lath-shaped crystals. No uncommon hazards were noted in all of the adopted synthetic procedures. Microanalytical data were obtained using a Fisons EA CHNS-O (Fisons, Loughborough, UK) instrument operating at 1000 °C. High-resolution positive ESI-MS spectra were acquired using an Orbitrap Elite mass spectrometer (Thermo Fisher Scientific, Waltham, MA, USA). Acetonitrile solutions were infused into the ESI source at a flow rate of 5.00 μL/min. Spectra were acquired in the *m*/*z* range 300−600 with a resolution of 240,000 (fwhm at *m*/*z* 400). Instrument settings were as follows: spray voltage, 3500 V; capillary temperature, 275 °C; probe heater temperature, 50 °C. Unfortunately, under the experimental conditions considered, the ESI-MS spectra recorded could not prove the structure of the products obtained, presumably due to reductive elimination of dihalogens. Raman spectroscopy experiments were performed in backscattered geometry with a nonconfocal micro-Raman OEM system. The emission at 785 nm from a fiber-coupled laser diode (BWTEK BRM-785) was focused onto the samples by means of a 10× microscope objective. Raman signals were recorded by a fiber-coupled grating spectrometer coupled with a Peltier-cooled CCD (BWTEK BTC667N-785S) with a spectral resolution of ≤ 5 cm^−1^. The values in parentheses next to the ν­(I−I) values represent the intensities of the bands relative to the strongest, taken equal to 10. The Rayleigh scattering was rejected by means of an edge-filter cutting at approximately 60 cm^−1^. Depending on the sample under investigation, the laser power was kept below 3 mW to avoid sample decomposition. The Raman spectra were recorded on the same crystals used for the X-ray diffraction studies.

### Synthesis

The hypercoordinate Te­(IV) compounds **L**I_2_ (**1**), **L**I_**2**_·1/2I_**2**_ (**2**), **L**Br_2_ (**3**), **L**Br_1.63_I_0.37_ (**4**), and **L**Cl_1.86_I_0.14_ (**5**) were obtained directly as crystals suitable for X-ray diffraction analysis. Due to the experimental synthetic procedures adopted, the reaction yields could not be determined.

#### *Synthesis of* **L**I_2_ (**1**)

A solution of I_2_ (10 μmol) in CHCl_3_ (2 mL) was added at room temperature to a solution of **L** (10 μmol) in CHCl_3_ (2 mL). The resulting mixture was stirred for 10 min at room temperature and then stored at −20 °C for 72 h, yielding red block-shaped crystals. Elem. Anal. Found (calc. for C_14_H_10_N_2_I_2_Te): C 28.23 (28.59); H 1.68 (1.71); N 4.55 (4.77). Raman ν (cm^−1^): 110 (10.0).

#### *Synthesis of* **L**I_2_·1/2I_2_ (**2**)

A solution of **L** (10 μmol) in CH_3_CN (2 mL) was carefully layered over a solution of I_2_ (20 μmol) in CHCl_3_ (1 mL). The system was left undisturbed at room temperature for 72 h, yielding orange needle-shaped crystals. Elem. Anal. Mp = 177 °C. Found (calcd for C_14_H_10_N_2_I_3_Te): C 23.35 (23.53); H 1.33 (1.41); N 3.82 (3.92). Raman ν (cm^−1^): 110 (7.7), 143 (2.2), 176 (10.0).

#### *Synthesis of* **L**Br_2_ (**3**)

A solution of Br_2_ (10 μmol) in CH_3_CN (2 mL) was added dropwise at room temperature to a solution of **L** (10 μmol) in CHCl_3_ (1 mL). The resulting mixture was stirred for 10 min at room temperature and then allowed to undergo slow partial evaporation at room temperature over 72 h, yielding orange lath-shaped crystals suitable for X-ray diffraction analysis. Mp = 204 °C. Elem. Anal. Found (calc. for C_14_H_10_N_2_Br_2_Te): C 34.00 (34.05); H 1.98 (2.04); N 5.73 (5.67). Raman ν (cm^−1^): 98 (1.6), 159 (10), 171sh (4.7).

#### *Synthesis of* **L**Br_1.63_I_0.37_ (**4**)

A solution of IBr (10 μmol) in CH_3_CN (2 mL) was added dropwise at room temperature to a solution of **L** (10 μmol) in CHCl_3_ (1 mL). The resulting mixture was stirred for 10 min at room temperature and then allowed to undergo slow partial evaporation at room temperature over 72 h, yielding red rod-shaped crystals suitable for X-ray diffraction analysis. Mp = 225 °C. Elem. Anal. Found (calc. for C_14_H_10_N_2_Br_1.63_I_0.37_Te): C 32.75 (32.90); H 2.12 (1.97); N 5.38 (5.48). Raman ν (cm^−1^): 105sh (1.9), 112 (6.6), 134 (10.0), 159 (8.9), 166sh (1.1).

#### *Synthesis of* **L**Cl_1.86_I_0.14_ (**5**)

A solution of ICl (10 μmol) in CH_3_CN (2 mL) was added dropwise at room temperature to a solution of **L** (10 μmol) in CHCl_3_ (1 mL). The resulting mixture was stirred for 10 min at room temperature and then allowed to undergo slow partial evaporation at room temperature over 72 h, yielding orange block-shaped crystals suitable for X-ray diffraction analysis. Mp = 220 °C. Elem. Anal. Found (calc. for C_14_H_10_N_2_Cl_1.86_I_0.14_Te): C 40.18 (40.27); H 2.29 (2.41); N 6.60 (6.71). Raman ν (cm^−1^): 105 (10.0), 148 (9.2), 213 (1.5), 266 (1.1).

### X-ray Structure Measurements

Single-crystal X-ray diffraction data were collected at 100 K on a Bruker D8 Venture diffractometer equipped with a PHOTON II detector. The structures were solved with the ShelXT^113^ solution program using dual methods and developed by iterative cycles of least-squares refinement on *F* ^2^ using ShelXL 2018/3.^114^ For compound **L**, all the tested crystals exhibited twinning, and the final model was built by refining the data as a two-component inversion twin. The disorder at both halogen sites in the crystal structures of hypercoordinate Te­(IV) compounds **L**Br_1.63_I_0.37_ (**4**) and **L**Cl_1.86_I_0.14_ (**5**) was investigated by carrying out a competitive refinement, with the mixed-site occupancy factors of the relevant halogen atoms adding up to one. Olex2 1.5 ^115^ and Mercury^116^ were used as the graphical interface and for the preparation of figures. Hydrogen atoms were placed geometrically and refined isotropically riding on their parent C atom, with *U*_iso_(H) = 1.2*U*_eq_(C).

### Computational Studies

The computational investigation was carried out at the DFT level^100^ by using the Gaussian 16 suite of programs.^117^ Basis sets were obtained from Basis Set Exchange and Basis Set EMSL Library.^118^ As a preliminary step, the computational setup was carefully validated. Over the past years,^80,119,120^ DFT calculations adopting the hybrid mPW1PW^121^ or the PBE0 ^122^ functionals paralleled by basis sets (BSs) belonging to the full-electron Ahlrichs Def2 basis set (BS) family^123^ were found to be in nice agreement with structural and spectroscopic experimental data involving chalcogen donors interacting with dihalogens/interhalogens. Moreover, several authors have reported on the excellent performance of the M06-2X functional,^124^ parametrized for nonmetals and including a double amount of nonlocal exchange as compared to M06,^125^ in modeling NCIs. Therefore, selected metric parameters and vibrational frequencies were calculated with the three functionals for compound **1**, chosen as a benchmark model, and compared to the relevant structural data. A comparison clearly shows that the three functionals behave closely for selected metric, vibrational, and thermochemical parameters, the mPW1PW functional providing the best agreement between the calculated and experimental Raman shift values corresponding to the stretching mode of the I−Te−I moiety in compound **1**. The BS effect was verified by comparing the results obtained with split valence and triple-ζ BSs for light elements, while a larger LANL08­(d)^126^ basis sets (BSs) with relativistic effective core potentials (RECPs) was used for heavier halogen and chalcogen atomic. The use of a larger BS did not result in significant variations in the calculated parameters: for example, the Te−I distances in compound **1** feature a very modest dependence (within 0.02 Å) on the computational setup (Table S10). Finally, since dispersion plays an important role in σ-hole interactions,^107^ the Grimme vdW dispersion energy-correction term^127^ was assessed for the PBE0 functional (PBE0-D3).^128^ A comparison of the results obtained with PBE0 and PBE0-D3 functional show only minor variations in metric parameters, vibrational frequencies, electronic and thermochemical energies, thus confirming the results previously obtained on different dibromine and diiodine adducts of a sulfur-rich thiocarbonyl donors.^119^ Since the BS effect and the dispersion corrections could be potentially more relevant when CT adducts are involved, also the CT model N-adduct **L**·I_2_ (see Discussion) was considered (Table S11). Although no experimental data are available for this CT adduct, also these calculations showed a negligible effect induced by the increase in the BS dimensions and the consideration of dispersion effects. Most importantly, all functional/BS combinations, either considering Grimme’s dispersion or not, show the same trend in the stability of CT and hypercoordinate compounds. Summarily, the mPW1PW functional paralleled by the Def2-SVP BS for light species and LANL08­(d) for chalcogens and halogen atomic species provides a computationally convenient, yet accurate, computational setup, that had been successfully used in the past in the investigation of the reactions of bis-4-imidazoline-2-selone derivatives and elemental diiodine.^63^ The memory required for each calculation was evaluated by the GaussMem cross-platform (Linux, macOS, Windows) program as a function of the number of shared processors, the total number of basis set functions, and a memory threshold depending on the highest angular momentum basis function.^80,129^ The molecular geometry optimizations were performed by starting from the available structural data. The nature of the minima of each optimized geometry was verified by harmonic frequency calculations. Charge distributions, Wiberg bond indexes,^103^ and intramolecular interactions were evaluated at the NBO level^69^ at the optimized geometries. The program GaussView 6.1 ^130^ was used to investigate the optimized structures, the isosurfaces of Kohn−Sham molecular orbitals, and the (molecular electrostatic potential) MEP maps.

## Conclusions

In this work, we have explored the reactivity of 2,5-bis­(pyridine-2-yl)­tellurophene (**L**) belonging to the class of π-conjugated tellurophenes (well-known for their optoelectronic properties and ability to form intermolecular interactions) toward elemental dihalogens XY (X = Y = I, Br, Cl; X = I, Y = Cl, Br). Compounds **L**I_2_ (**1**), **L**I_2_·1/2I_2_ (**2**), **L**Br_2_ (**3**), **L**Br_1.63_I_0.37_ (**4**), and **L**Cl_1.86_I_0.14_ (**5**) were isolated and characterized in the solid state. In all cases, the oxidation of the Te­(II) center in **L** is observed with the formation of hypercoordinate Te­(IV) adducts featuring three center-four electron X−Te−X moieties and a seesaw geometry at the chalcogen atom. Notably, compounds **1** and **2** represent the first examples of an I_2_ oxidative addition to a tellurophene ring. A comparative analysis of the structural features of the compounds obtained allowed to rationalize the influence of the halogen atom on the type of intermolecular noncovalent interactions (NCIs: HaBs and ChBs).

In fact, structural analysis reveals that the interplay between halogen and chalcogen bonding dictates the supramolecular organization, significantly influenced by the periplanar conformation of **L** enforced by the ortho-pyridyl substituents. Theoretical calculations further support the observed electronic and structural properties, shedding light on the charge distribution and bond polarization trends.

In this respect, we observed that among the “T-shaped” dihalogen adducts presented in this study, the largest anisotropic MEP calculated for iodinated compounds (**1** and **2**) favor the formation of multiple and synergistic NCIs, including type I and type II HaBs, as well as nonclassical ChBs, which are embedded in the pseudosquare Te_2_I_2_ motifs.

A systematic comparison with structurally related dihalogen adducts of π-conjugated tellurophene reveals that excessive steric hindrance hampers the formation of intermolecular NCIs, particularly HaBs, whereas ChBs are preserved in some cases, especially in nonplanar environments.

From a crystal engineering perspective, the consistent and predictable formation of seesaw, rather than CT ″spoke″ adducts, in this family of compounds is clearly corroborated by both experimental and theoretical evidence. These findings provide valuable insights into the design of tellurophene-based materials with tailored supramolecular and electronic properties, paving the way for their potential applications in optoelectronics and crystal engineering.

## Supplementary Material



## Notes

View of the asymmetric unit in the X-ray crystal structure of compound **L** (Figure S1); view of the crystal packing in **L** (Figure S2); views of the crystal packing in **L**I_2_ (**1**) (Figure S3); asymmetric units for X-ray crystal structure of **L**Br_1.63_I_0.37_ (**4**) and **L**Cl_1.86_I_0.14_ (**5**) (Figure S4); Te···Br interactions and crystal packing in **L** ^**c**^Br_2_ (Figure S5); Te···X interactions and crystal packing in **L** ^**f**^X_2_ (X=Br, Cl; CCDC codes: HAPKEQ and PIVVOH, respectively) (Figure S6); Venn diagram resulting from a CCDC search showing the classification of halogenated hypercoordinate chalcogen derivatives featuring coordination number (CN) = 4 and only two halogen atoms bound to the chalcogen (Figure S7); distribution of the β angle (calculated between the two planes defined by the fragments X−Ch−X and E−Ch−E) for the structures belonging to Group 1 in the Venn diagram (Figure S8); distribution of compounds in Group 1 in the Venn diagram based on the nature of the chalcogen and halogen atoms (Figure S9); correlation between the charge-transfer from the donor moiety to the coordinated dihalogen molecule calculated at DFT level and the relative interhalogen bond length in **L**·XY CT-adducts at the tellurium atom (XY = Cl_2_, Br_2_, I_2_, ICl, and IBr) (Figure S10); crystal structure and refinement parameters for: **L**, **L**I_2_ (**1**), **L**I_2_·1/2I_2_ (**2**), **L**Br_2_ (**3**), **L**Br_1.63_I_0.37_ (**4**) and **L**Cl_1.86_I_0.14_ (**5**) (Table S1); bond lengths (Å) and angles (°) for: **L**, **L**I_2_ (**1**), **L**I_2_·1/2I_2_ (**2**), **L**Br_2_ (**3**), **L**Br_1.63_I_0.37_ (**4**), and **L**Cl_1.86_I_0.14_ (**5**) (Tables S2−S7); selected parameters calculated for the CT-adducts **L**·XY interacting via either the tellurium or the nitrogen donor atoms (Table S8); thermodynamic parameters calculated in the gas phase for **L** and corresponding 1:1 XY adducts (Table S9); selected experimental and calculated structural metric parameters, energies, and Raman shift values for compound **L**I_2_ (**1**) (Table S10), and for the model CT N-adduct **L**·I_2_ (Table S11) considered for the validation of the computational setup (PDF)
