# Supplementary material for: Synergistic Interplay between Intermolecular Halogen and Chalcogen Bonds in the Dihalogen Adducts of 2,5-Bis(pyridine-2-yl)tellurophene: Reactivity Insights and Structural Trends
Source: Inorg Chem. 2025 May 29;64(22):10972–88. doi: 10.1021/acs.inorgchem.5c01084 (PMC12152948; doi:10.1021/acs.inorgchem.5c01084)
Supplement: Supplementary file 1 [file ic5c01084_si_001.pdf]

Supporting Information

Synergistic Interplay Between Intermolecular Halogen and Chalcogen Bonds in the Dihalogen Adducts of 2,5-Bis(pyridine-2-yl)tellurophene: Reactivity Insights and Structural Trends

Enrico Podda[a], Massimiliano Arca[b], Maria Carla Aragoni[b], Claudia Caltagirone[b], Vito Lippolis[b]*, Anna Pintus[b], Douglas B. Paixão[c], Eduardo G. O. Soares[c], Paulo H. Schneider[c]

[a] Centro Servizi di Ateneo per la Ricerca (CeSAR), Università degli Studi di Cagliari, S.S. 554 Bivio per Sestu, 09042 Monserrato (CA), Italy

[b] Dipartimento di Scienze Chimiche e Geologiche, Università degli Studi di Cagliari, S.S. 554 Bivio per Sestu, 09042 Monserrato (CA), Italy

[c] Institute of Chemistry, Federal University of Rio Grande do Sul (UFRGS), Av. Bento Gonçalves 9500, 91501-970 P. O. Box: 15003, Porto Alegre-RS, Brazil

* Email: lippolis@unica.it

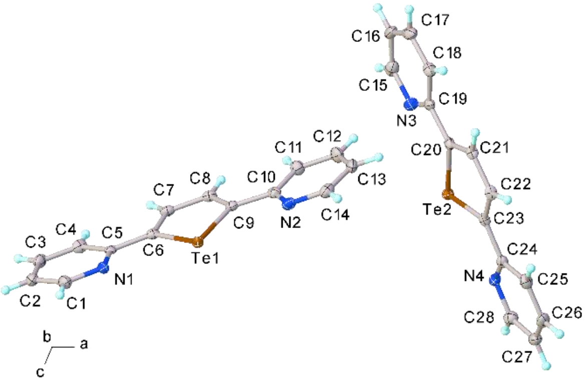

Figure S1. View along the *b*-axis of the asymmetric unit in the X-ray crystal structure of compound **L**. The dihedral angle between the planes determined by each independent unit is 82°. Thermal ellipsoids are drawn at 50% probability level.

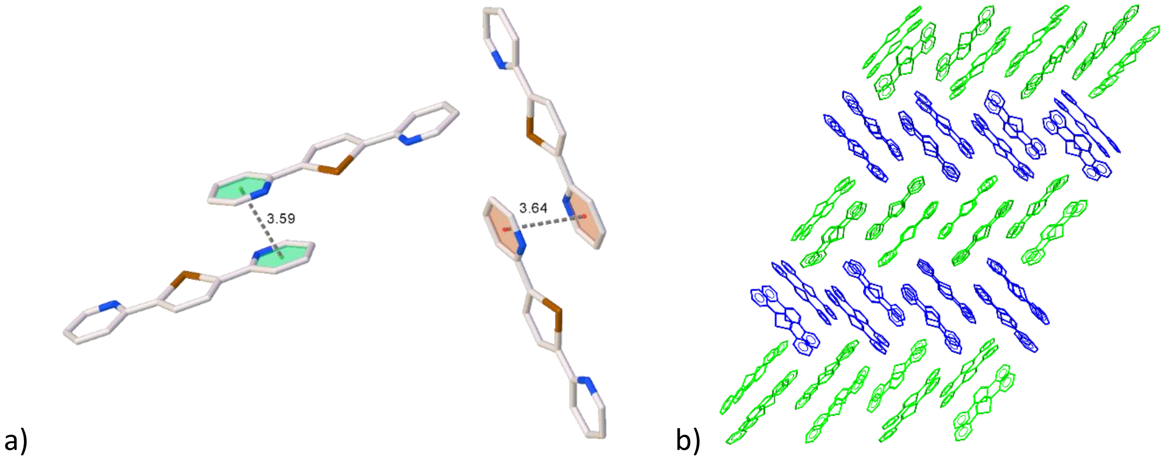

Figure S2. π-π Stacking intermolecular interactions between symmetry-related units of **L** and selected inter-centroid distances (Å) (a); perspective view of the crystal packing of **L** along the *b*-axis, with molecules colored according to their symmetry-based equivalence (b).

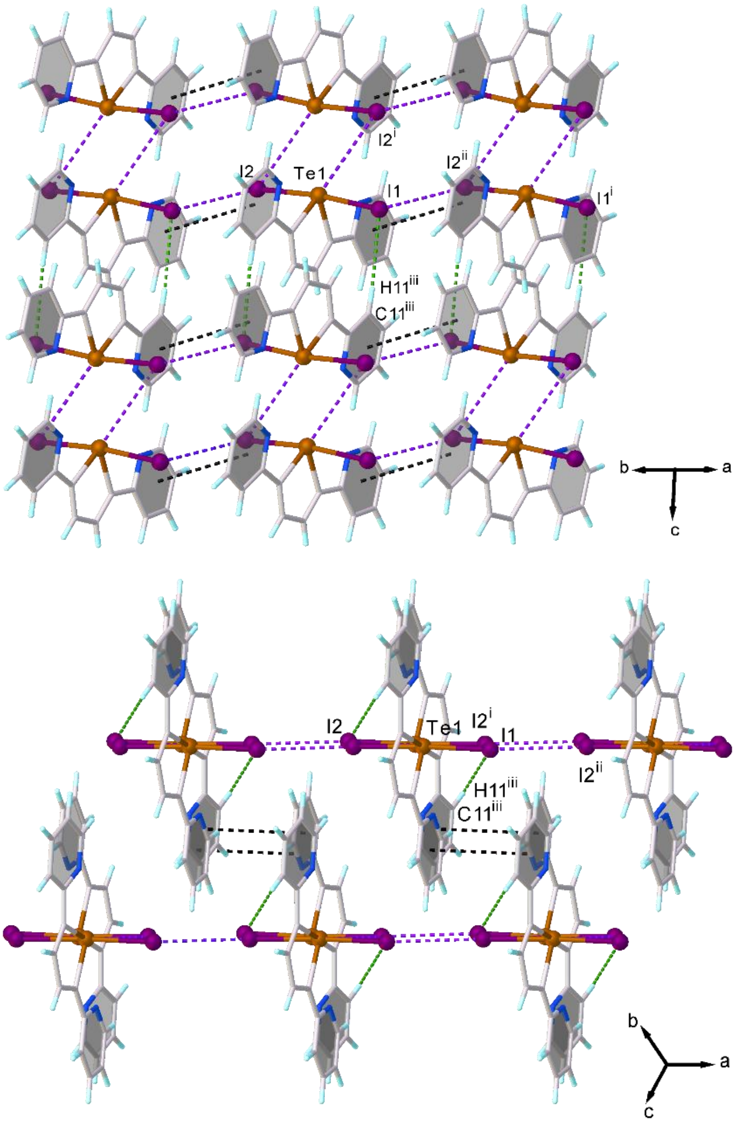

Figure S3. Alternative views of the crystal packing in **L**I$_2$ (**1**) showing slipped π-π stacking interactions between pyridyl rings (dihedral angle between pyridyl rings 10°, inter-centroid distance 3.95 Å, shift distance 1.63 Å).

	**L**Cl$_{1.86}$I$_{0.14}$ (**5**)		**L**Br$_{1.63}$I$_{0.37}$ (**4**)	
	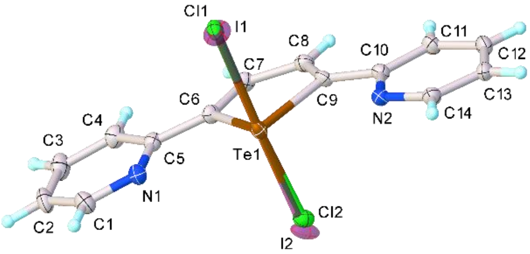		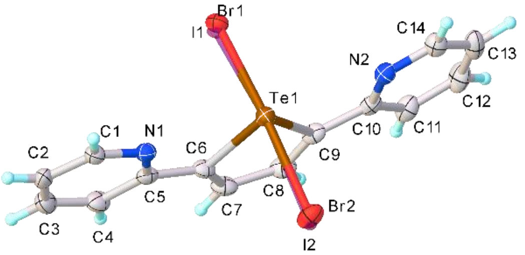	
Atom (*sof**)	Cl1 (0.91)	I1 (0.09)	Br1 (0.85)	I1 (0.15)
	Cl2 (0.95)	I2 (0.05)	Br2 (0.78)	I2 (0.22)
**sof* = site occupancy factor				

Figure S4. X-ray crystal structure of **L**Br$_{1.63}$I$_{0.37}$ (**4**, right) and **L**Cl$_{1.86}$I$_{0.14}$ (**5**, right) with indications of site occupancy factors, and atom labelling scheme adopted. Thermal ellipsoids are drawn at 50% probability level.

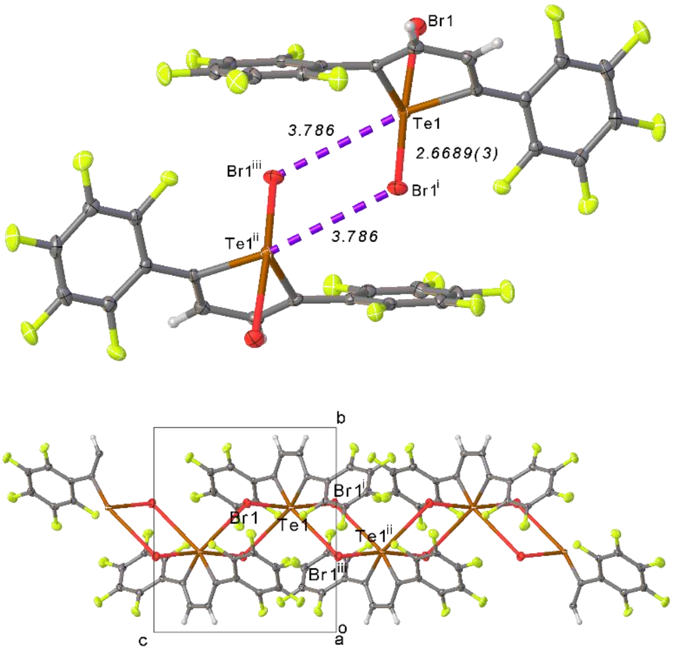

Figure S5. Te···Br interactions and crystal packing in **L**^{c}Br$_2$ (^{i} = 1-x, +y, ½-z; ^{ii} = 1-x, 1-y, -z; ^{iii} = +x, 1-y, ½+z); see ref. 55 in the main text (CCDC code: EHUKOJ).

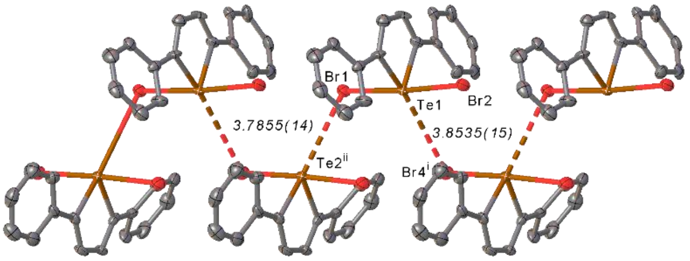

^{i} = 1+X,+Y,1+Z ^{ii} = +X,+Y,1+Z

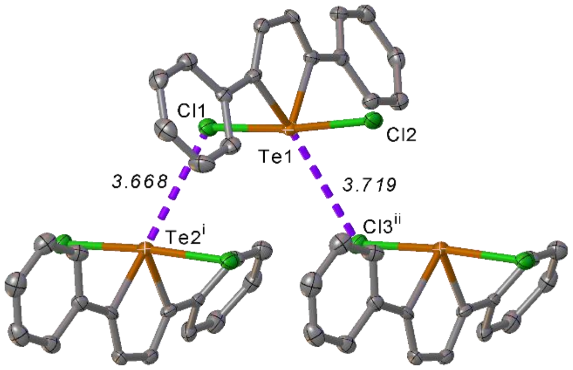

^{i} = +X,1-Y,1/2+Z ^{ii} = 1+X,1-Y,1/2+Z

Figure S6. Te···X interactions and crystal packing in **L**^{f}X$_2$ (X = Br, Cl; CCDC codes: HAPKEQ and PIVVOH, respectively); see ref. 56 in the main text.

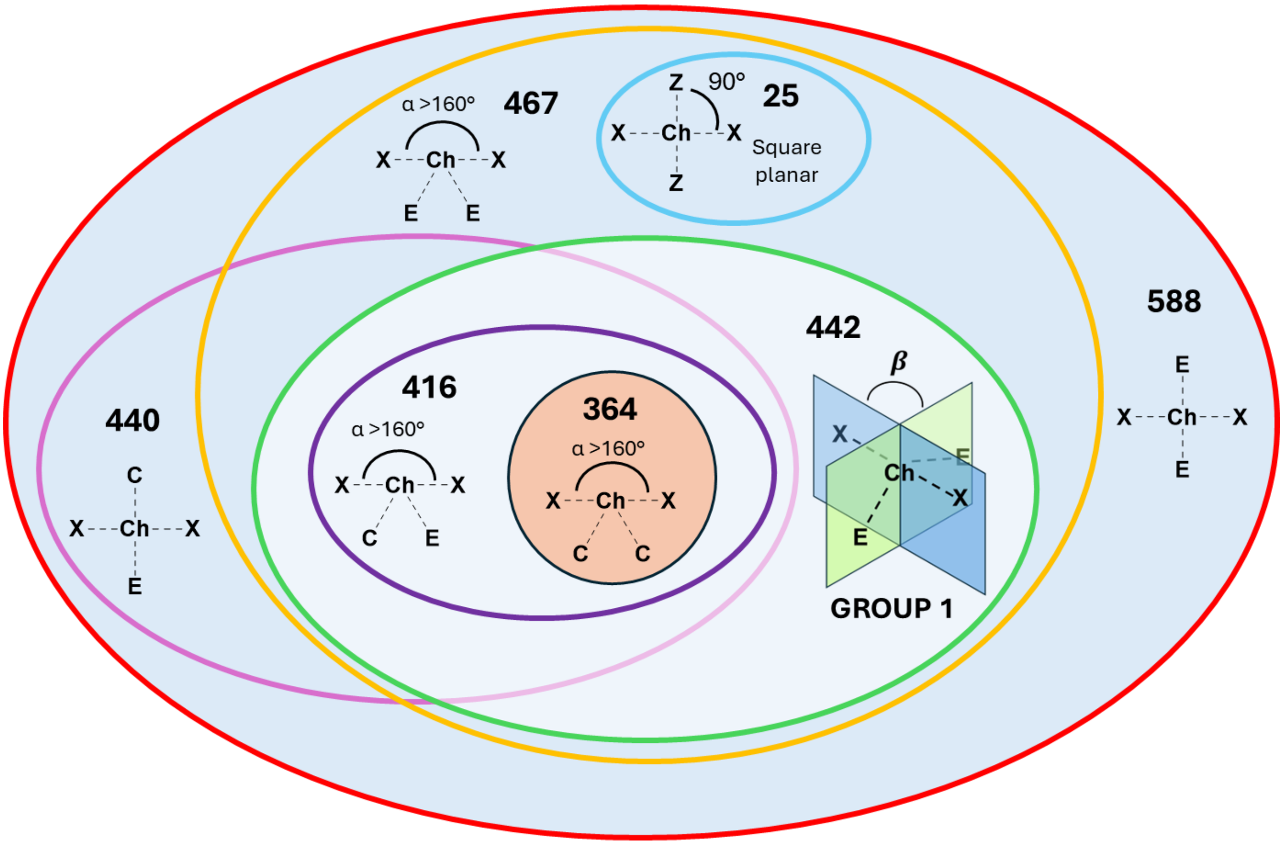

Figure S7. Venn diagram resulting from a CCDC search (4th Feb 2025; ConQuest Version 2024.3.0) showing the classification of halogenated hypercoordinate chalcogen derivatives featuring coordination number (CN) = 4 and only two halogen atoms bound to the chalcogen. Ch = S, Se, Te with CN = 4; X = F, Cl, Br, I; E = any element different from halogen. The 25 hits featuring a square planar geometry are characterized by a central chalcogen(II) atom and Z = S, Se.

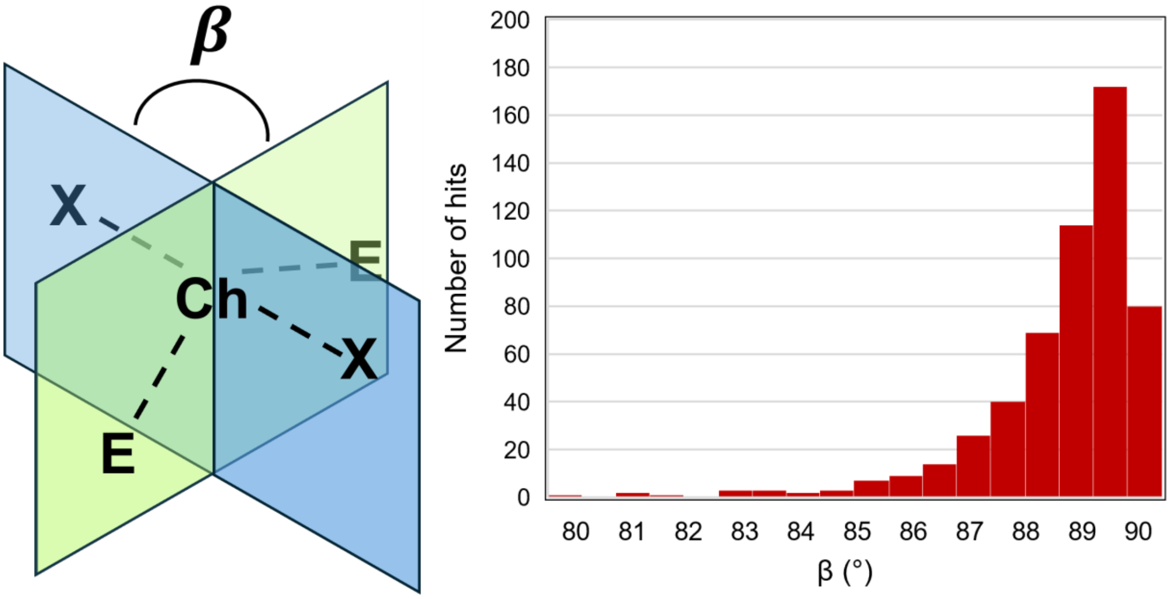

Figure S8. Distribution of the β angle (calculated between the two planes defined by the fragments X–Ch–X and E–Ch–E) for the structures belonging to Group 1 in Figure S7. Only for two compounds (CCDC codes: INESAS and EHOLOF) a β angle lower than 80° (75° and 73°, respectively) is observed.

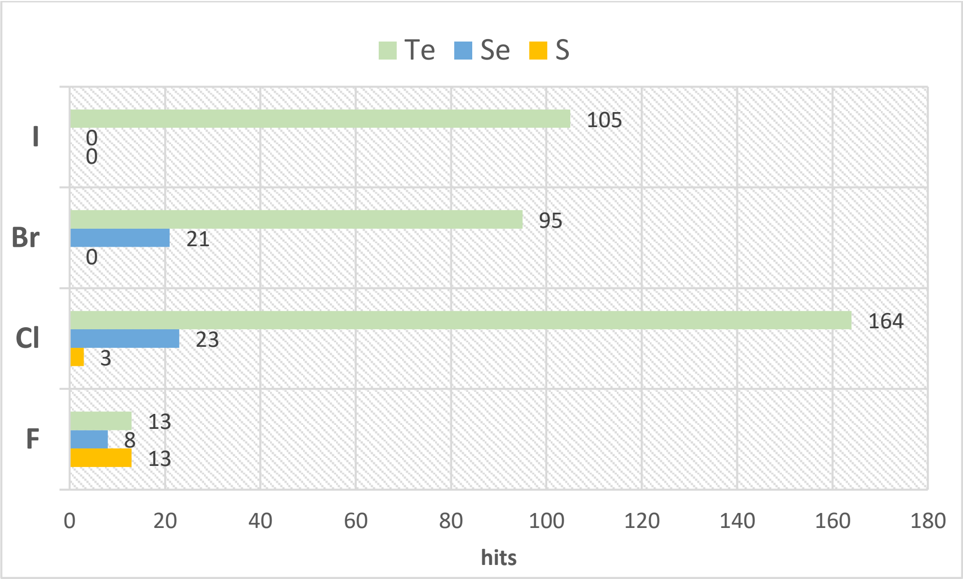

Figure S9. Distribution of Group 1 (Figure S7) based on the nature of the chalcogen and halogen atoms. The number of hits refers to systems having at least one I, Br, Cl, or F in the linear X–Ch–X fragment.

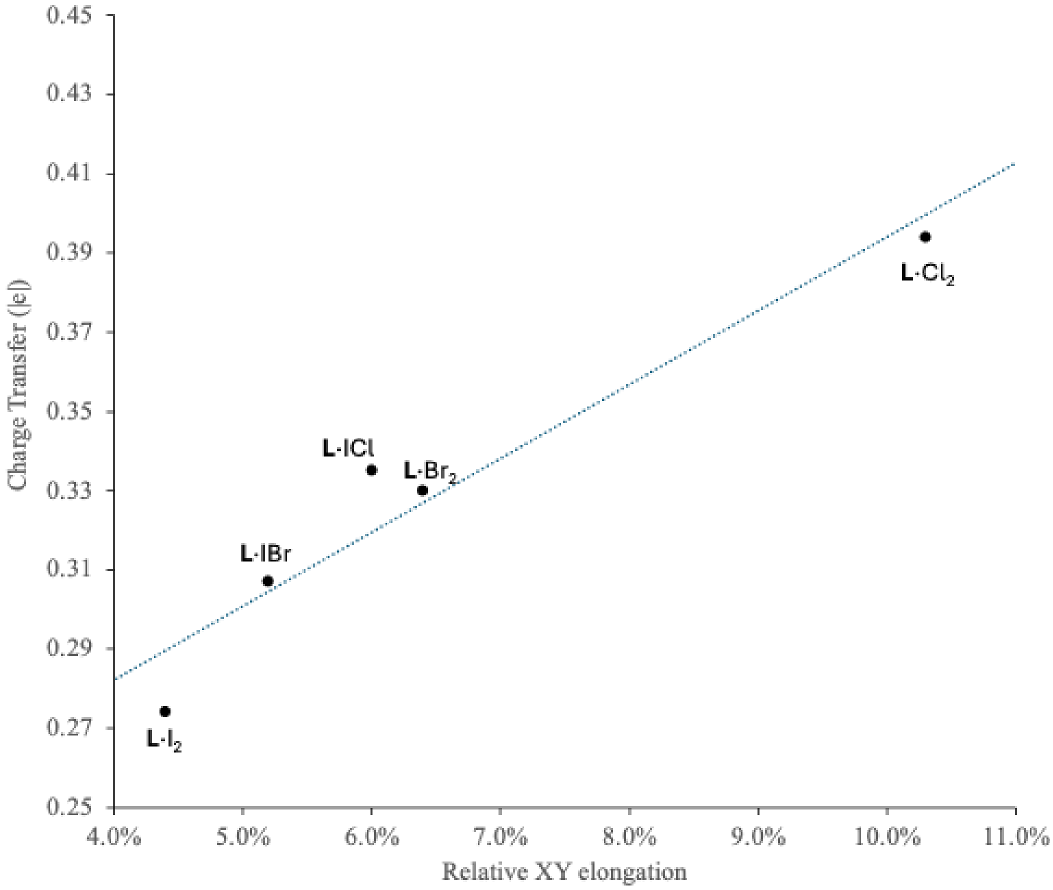

Figure S10. Correlation between the charge-transfer from the donor moiety to the coordinated dihalogen molecule calculated at DFT level and the relative interhalogen bond length in **L**·XY CT-adducts at the tellurium atom (XY = Cl_2, Br_2, I_2, ICl, and IBr; coefficient of determination R^2 = 0.93).

Table S1. Crystal structure and refinement parameters for: **L**, **L**I_2 (**1**), **L**I_2·1/2I_2 (**2**), **L**Br_2 (**3**), **L**$Br_{1.63}I_{0.37}$ (**4**) and **L**$Cl_{1.86}I_{0.14}$ (**5**).

Compound	**L**	**L**I_2	**L**I_2·1/2I_2	**L**Br_2	**L**$Cl_{1.86}I_{0.14}$	**L**$Br_{1.63}I_{0.37}$
Empirical formula	$C_{14}H_{10}N_2Te$	$C_{14}H_{10}I_2N_2Te$	$C_{14}H_{10}I_3N_2Te$	$C_{14}H_{10}Br_2N_2Te$	$C_{14}H_{10}Cl_{1.86}I_{0.14}N_2Te$	$C_{14}H_{10}Br_{1.63}I_{0.37}N_2Te$
Formula weight	333.84	587.64	714.54	493.66	417.77	511.52
Temperature/K	100(2)	100(2)	100(2)	100(2)	100(2)	100(2)
Crystal system	monoclinic	triclinic	triclinic	monoclinic	monoclinic	monoclinic
Space group	$P2_1/c$	$P\bar{1}$	$P\bar{1}$	$P2_1/n$	$P2_1/n$	$P2_1/n$
a/Å	23.691(7)	9.3451(8)	7.9317(15)	9.7367(7)	9.6154(12)	9.8263(7)
b/Å	5.9501(16)	9.6463(8)	10.5275(19)	11.0919(9)	10.8899(14)	11.1353(9)
c/Å	18.698(5)	10.1521(9)	11.043(2)	13.8312(12)	13.6857(18)	13.9466(11)
α/°	90	98.309(3)	75.750(8)	90	90	90
β/°	113.077(14)	102.607(3)	86.357(8)	99.806(3)	98.533(5)	100.582(3)
γ/°	90	112.898(3)	73.753(8)	90	90	90
Volume/Å^3	2424.8(12)	795.48(12)	858.1(3)	1471.9(2)	1417.2(3)	1500.1(2)
Z	8	2	2	4	4	4
ρ_{calc}/g cm^{-3}	1.829	2.453	2.766	2.228	1.958	2.265
μ/mm^{-1}	2.430	5.739	7.122	7.437	2.746	7.067
Crystal size/mm^3	0.32 × 0.20 × 0.08	0.19 × 0.13 × 0.08	0.38 × 0.01 × 0.01	0.42 × 0.17 × 0.16	0.36 × 0.30 × 0.09	0.31 × 0.11 × 0.10
Radiation	Mo*K*α (λ = 0.71073)	Mo*K*α (λ = 0.71073)	Mo*K*α (λ = 0.71073)	Mo*K*α (λ = 0.71073)	Mo*K*α (λ = 0.71073)	Mo*K*α (λ = 0.71073)
2θ range for data collection/°	3.544 to 50.7	4.252 to 55.202	3.806 to 50.294	4.734 to 57.552	4.8 to 59.328	4.712 to 59.256
Reflections collected	50076	49902	13415	34055	30315	34790
Independent reflections	4443 [R_{int} = 0.0503, R_{sigma} = 0.0227]	3676 [R_{int} = 0.0639, R_{sigma} = 0.0238]	3077 [R_{int} = 0.1152, R_{sigma} = 0.0848]	3811 [R_{int} = 0.0550, R_{sigma} = 0.0303]	3999 [R_{int} = 0.0532, R_{sigma} = 0.0334]	4240 [R_{int} = 0.0588, R_{sigma} = 0.0316]
Data/restraints/parameters	4443/0/308	3676/0/172	3077/24/182	3811/0/173	3999/0/192	4240/0/192
Goodness-of-fit on F^2	1.117	1.060	1.010	1.078	1.101	1.178
Final R indexes [I ≥ 2σ (I)]	R_1 = 0.0279, wR_2 = 0.0747	R_1 = 0.0186, wR_2 = 0.0400	R_1 = 0.0495, wR_2 = 0.1152	R_1 = 0.0194, wR_2 = 0.0471	R_1 = 0.0270, wR_2 = 0.0530	R_1 = 0.0296, wR_2 = 0.0631
Final R indexes [all data]	R_1 = 0.0281, wR_2 = 0.0749	R_1 = 0.0242, wR_2 = 0.0427	R_1 = 0.0751, wR_2 = 0.1286	R_1 = 0.0212, wR_2 = 0.0478	R_1 = 0.0360, wR_2 = 0.0556	R_1 = 0.0350, wR_2 = 0.0647
Largest diff. peak/hole / e Å^{-3}	2.68/−0.79	0.67/−0.64	1.89/−1.83	0.81/−0.52	0.55/−0.84	1.15/−0.88

Table S2. Bond lengths (Å) and angles (°) for **L**.

Te1–C6	2.070(5)	C3–C4	1.388(7)
Te1–C9	2.073(5)	C7–C8	1.426(7)
Te2–C20	2.070(5)	C8–C9	1.364(7)
Te2–C23	2.071(4)	C9–C10	1.466(7)
N2–C10	1.348(7)	C10–C11	1.398(7)
N2–C14	1.344(7)	C14–C13	1.387(8)
C22–C21	1.426(6)	C13–C12	1.383(8)
C22–C23	1.358(6)	C12–C11	1.388(7)
N4–C24	1.330(7)	C20–C21	1.365(7)
N4–C28	1.349(7)	C20–C19	1.471(6)
N3–C19	1.354(6)	C23–C24	1.471(6)
N3–C15	1.347(6)	C24–C25	1.409(7)
C6–C5	1.462(6)	C28–C27	1.387(7)
C6–C7	1.358(7)	C27–C26	1.385(8)
C5–N1	1.336(6)	C26–C25	1.389(7)
C5–C4	1.403(6)	C19–C18	1.406(7)
N1–C1	1.343(6)	C15–C16	1.393(8)
C1–C2	1.387(7)	C16–C17	1.385(9)
C2–C3	1.391(7)	C17–C18	1.393(7)
C6–Te1–C9	81.27(18)	N2–C14–C13	123.6(5)
C20–Te2–C23	81.41(18)	C12–C13–C14	118.4(5)
C14–N2–C10	117.9(4)	C13–C12–C11	118.9(5)
C23–C22–C21	117.7(4)	C12–C11–C10	119.4(5)
C24–N4–C28	118.5(4)	C21–C20–Te2	111.1(3)
C15–N3–C19	117.0(4)	C21–C20–C19	128.8(4)
C5–C6–Te1	120.1(3)	C19–C20–Te2	120.0(3)
C7–C6–Te1	111.5(3)	C20–C21–C22	118.1(4)
C7–C6–C5	128.2(4)	C22–C23–Te2	111.6(3)
N1–C5–C6	116.0(4)	C22–C23–C24	128.5(4)
N1–C5–C4	121.6(4)	C24–C23–Te2	119.7(3)
C4–C5–C6	122.4(4)	N4–C24–C23	115.9(4)
C5–N1–C1	119.0(4)	N4–C24–C25	122.0(4)
N1–C1–C2	123.2(5)	C25–C24–C23	122.2(4)
C1–C2–C3	118.0(5)	N4–C28–C27	123.3(5)
C4–C3–C2	119.2(5)	C26–C27–C28	118.1(5)
C3–C4–C5	119.0(5)	C27–C26–C25	119.4(5)
C6–C7–C8	118.1(4)	C26–C25–C24	118.7(5)
C9–C8–C7	117.6(4)	N3–C19–C20	115.7(4)
C8–C9–Te1	111.4(3)	N3–C19–C18	122.7(4)
C8–C9–C10	127.8(4)	C18–C19–C20	121.6(5)
C10–C9–Te1	120.7(3)	N3–C15–C16	124.2(5)
N2–C10–C9	116.8(4)	C17–C16–C15	118.1(5)
N2–C10–C11	121.8(4)	C16–C17–C18	119.4(5)
C11–C10–C9	121.4(4)	C17–C18–C19	118.6(5)

Table S3. Bond lengths (Å) and angles (°) for **L**I_2 (**1**).

I1–Te1	2.9223(4)	C4–C5	1.390(4)
I2–Te1	2.8961(4)	C5–C6	1.446(4)
Te1–C6	2.108(3)	C6–C7	1.346(4)
Te1–C9	2.116(3)	C7–C8	1.443(4)
N1–C1	1.333(4)	C8–C9	1.341(4)
N1–C5	1.348(4)	C9–C10	1.450(4)
N2–C10	1.347(4)	C10–C11	1.390(4)
N2–C14	1.334(4)	C11–C12	1.382(4)
C1–C2	1.380(5)	C12–C13	1.384(4)
C2–C3	1.379(5)	C13–C14	1.381(4)
C3–C4	1.384(4)		
I2–Te1–I1	174.244(8)	C5–C6–Te1	115.61(19)
C6–Te1–I1	88.28(8)	C7–C6–Te1	111.6(2)
C6–Te1–I2	87.38(8)	C7–C6–C5	132.7(3)
C6–Te1–C9	80.15(11)	C6–C7–C8	118.3(3)
C9–Te1–I1	87.46(8)	C9–C8–C7	118.4(2)
C9–Te1–I2	88.07(8)	C8–C9–Te1	111.5(2)
C1–N1–C5	117.8(3)	C8–C9–C10	133.4(3)
C14–N2–C10	117.6(3)	C10–C9–Te1	114.97(19)
N1–C1–C2	123.3(3)	N2–C10–C9	114.0(2)
C3–C2–C1	118.7(3)	N2–C10–C11	122.6(3)
C2–C3–C4	119.1(3)	C11–C10–C9	123.4(3)
C3–C4–C5	118.5(3)	C12–C11–C10	118.8(3)
N1–C5–C4	122.5(3)	C11–C12–C13	119.0(3)
N1–C5–C6	113.9(3)	C14–C13–C12	118.6(3)
C4– C5–C6	123.7(3)	N2– C14–C13	123.5(3)

Table S4. Bond lengths (Å) and angles (°) for **L**I_2·½I_2 (**2**).

Te1–I2	2.9511(12)	C9–C8	1.339(17)
Te1–I1	2.8750(12)	C6–C7	1.340(17)
Te1–C9	2.120(12)	C6–C5	1.458(17)
Te1–C6	2.121(11)	C7–C8	1.436(17)
I3–I3^{i}	2.7326(18)	C2–C1	1.377(17)
N2–C10	1.374(15)	C2–C3	1.401(18)
N2–C14	1.343(15)	C14–C13	1.384(17)
N1–C1	1.353(15)	C13–C12	1.386(18)
N1–C5	1.319(15)	C11–C12	1.341(18)
C10–C9	1.439(17)	C3–C4	1.369(18)
C10–C11	1.363(17)	C5–C4	1.396(17)
I1–Te1–I2	175.39(4)	C7–C6–C5	132.1(11)
C9–Te1–I2	87.1(3)	C5–C6–Te1	115.8(8)
C9–Te1–I1	88.6(3)	C6–C7–C8	117.8(11)
C9–Te1–C6	79.5(5)	C1–C2–C3	118.9(12)
C6–Te1–I2	88.6(3)	N1–C1–C2	122.3(11)
C6–Te1–I1	89.1(3)	N2–C14–C13	122.9(11)
C14–N2–C10	117.7(10)	C14–C13–C12	117.3(11)
C5–N1–C1	117.7(10)	C12–C11–C10	120.7(12)
N2–C10–C9	112.5(10)	C11–C12–C13	120.2(12)
C11–C10–N2	121.0(11)	C4–C3–C2	119.1(12)
C11–C10–C9	126.4(11)	C9–C8–C7	119.1(12)
C10–C9–Te1	114.7(8)	N1–C5–C6	114.1(11)
C8–C9–Te1	111.4(9)	N1–C5–C4	124.1(12)
C8–C9–C10	133.9(12)	C4–C5–C6	121.8(11)
C7–C6–Te1	112.1(9)	C3–C4–C5	117.9(12)

Symmetry code: ^{i}1−*x*, 2−*y*, −*z*.

Table S5. Bond lengths (Å) and angles (°) for **L**Br_2 (**3**).

Te1–Br1	2.6482(3)	C4–C5	1.394(3)
Te1–Br2	2.6690(3)	C5–C6	1.454(3)
Te1–C6	2.110(2)	C6–C7	1.344(3)
Te1–C9	2.1076(19)	C7–C8	1.457(3)
N1–C1	1.334(3)	C8–C9	1.338(3)
N1–C5	1.348(3)	C9–C10	1.447(3)
N2–C10	1.345(3)	C10–C11	1.399(3)
N2–C14	1.340(3)	C11–C12	1.384(3)
C1–C2	1.386(3)	C12–C13	1.384(4)
C2–C3	1.381(3)	C13–C14	1.384(3)
C3–C4	1.381(3)		
Br1–Te1–Br2	172.585(8)	C5–C6–Te1	114.49(14)
C6–Te1–Br1	85.52(6)	C7–C6–Te1	111.66(15)
C6–Te1–Br2	87.95(6)	C7–C6–C5	133.6(2)
C9–Te1–Br1	86.65(6)	C6–C7–C8	117.64(19)
C9–Te1–Br2	88.82(6)	C9–C8–C7	118.84(19)
C9–Te1–C6	80.53(8)	C8–C9–Te1	111.32(15)
C1–N1–C5	117.54(18)	C8–C9–C10	133.53(19)
C14–N2–C10	117.92(18)	C10–C9–Te1	114.99(14)
N1–C1–C2	123.5(2)	N2–C10–C9	114.17(17)
C3–C2–C1	118.56(19)	N2–C10–C11	122.6(2)
C4–C3–C2	119.1(2)	C11–C10–C9	123.2(2)
C3–C4–C5	118.7(2)	C12–C11–C11	118.3(2)
N1–C5–C4	122.58(19)	C11–C12–C13	119.4(2)
N1–C5–C6	113.27(18)	C14–C13–C12	118.6(2)
C4–C5–C6	124.09(19)	N2–C14–C13	123.2(2)

Table S6. Bond lengths (Å) and angles (°) for **L**$Br_{1.63}I_{0.37}$ (**4**).

Te1–Br2	2.717(4)	C10–C11	1.400(5)
Te1–Br1	2.731(4)	C1–C2	1.388(5)
Te1–C9	2.108(3)	C11–C12	1.380(6)
Te1–C6	2.107(3)	C2–C3	1.383(6)
Te1–I2	2.763(8)	C8–C7	1.450(6)
Te1–I1	2.578(16)	C6–C5	1.457(5)
N2–C10	1.345(5)	C6–C7	1.337(5)
N2–C14	1.329(5)	C5–C4	1.397(5)
N1–C1	1.331(5)	C4–C3	1.372(6)
N1–C5	1.343(5)	C12–C13	1.376(7)
C9–C10	1.448(5)	C14–C13	1.393(6)
C9–C8	1.340(5)		
Br2–Te1–Br1	174.16(13)	C11–C10–C9	123.0(4)
C9–Te1–Br2	89.21(12)	N1–C1–C2	124.1(4)
C9–Te1–Br1	87.17(14)	C12–C11–C10	117.9(4)
C9–Te1–I2	88.52(18)	C3–C2–C1	117.8(4)
C9–Te1–I1	86.6(4)	C9–C8–C7	118.9(3)
C6–Te1–Br2	88.54(12)	C5–C6–Te1	115.0(2)
C6–Te1–Br1	86.35(14)	C7–C6–Te1	111.8(3)
C6–Te1–C9	80.43(15)	C7–C6–C5	133.0(4)
C6–Te1–I2	86.82(16)	N1–C5–C6	113.5(3)
C6–Te1–I1	83.5(4)	N1–C5–C4	122.5(4)
I1–Te1–I2	169.7(4)	C4–C5–C6	123.9(4)
C14–N2–C10	118.0(3)	C3–C4–C5	118.8(4)
C1–N1–C5	117.3(3)	C13–C12–C11	119.9(4)
C10–C9–Te1	115.3(3)	C6–C7–C8	117.8(3)
C8–C9–Te1	111.1(3)	N2–C14–C13	123.1(4)
C8–C9–C10	133.5(3)	C4–C3–C2	119.4(4)
N2–C10–C9	114.3(3)	C12–C13–C14	118.3(4)
N2–C10–C11	122.7(4)		

Table S7. Bond lengths (Å) and angles (°) for **L**$Cl_{1.86}I_{0.14}$ (**5**).

Te1–Cl1	2.576(5)	C10–C11	1.392(4)
Te1–Cl2	2.500(3)	C10–C9	1.457(4)
Te1–C9	2.111(2)	C8–C7	1.448(4)
Te1–C6	2.103(2)	C8–C9	1.339(4)
Te1–I1	2.525(13)	C12–C11	1.382(4)
Te1–I2	2.77(2)	C12–C13	1.384(4)
N2–C10	1.351(3)	C4–C3	1.380(4)
N2–C14	1.334(3)	C7–C6	1.346(4)
N1–C5	1.347(3)	C3–C2	1.384(4)
N1–C1	1.332(4)	C2–C1	1.387(4)
C5–C4	1.393(4)	C14–C13	1.388(4)
C5–C6	1.446(4)		
Cl2–Te1–Cl1	172.15(13)	C11–C10–C9	124.1(2)
C9–Te1–Cl1	87.99(11)	C9–C8–C7	117.9(2)
C9–Te1–Cl2	85.65(10)	C11–C12–C13	119.3(3)
C9–Te1–I1	88.3(3)	C12–C11–C10	118.7(2)
C9–Te1–I2	88.0(4)	C3–C4–C5	118.7(3)
C6–Te1–Cl1	88.05(13)	C6–C7–C8	118.9(2)
C6–Te1–Cl2	86.38(12)	C4–C3–C2	119.1(3)
C6–Te1–C9	80.60(10)	C3–C2–C1	118.4(3)
C6–Te1–I1	89.2(3)	N1–C1–C2	123.4(3)
C6–Te1–I2	86.8(5)	C10–C9–Te1	114.33(17)
I1–Te1–I2	175.0(5)	C8–C9–Te1	111.55(19)
C14–N2–C10	117.5(2)	C8–C9–C10	133.8(2)
C1–N1–C5	117.7(2)	N2–C14–C13	123.5(2)
N1–C5–C4	122.6(3)	C12–C13–C14	118.4(2)
N1–C5–C6	113.9(2)	C5–C6–Te1	115.53(18)
C4–C5–C6	123.5(2)	C7–C6–Te1	111.00(19)
N2–C10–C11	122.6(2)	C7–C6–C5	133.3(2)
N2–C10–C9	113.3(2)		

Table S8. Selected optimized distances d (Å) and relative dihalogen elongation, natural charge on the donor atom ($Q_{Te/N}$, |e|) and on the halogen atoms (Q_X and Q_Y, |e|), charge separation in the dihalogen unit XY ($|\Delta Q_{XY}|$,|e|), charge-transfer from the donor **L** to the dihalogen unit XY (|CT|,|e|), and relevant Wiberg bond indexes (WBI) calculated for the CT-adducts **L**·XY interacting via either the tellurium or the nitrogen donor atoms.

Donor atom	**XY**	$d_{Te/N–X}$	$d_{X–Y}$	$\Delta d_{XY}/d_{XY}$	$Q_{Te/N}$	Q_X	Q_Y	$\|\Delta Q_{XY}\|$	**|CT|**	$WBI_{Te/N\cdots X}$	$WBI_{X–Y}$
Te	Cl_2	2.790	2.218	10.3%	1.124	–0.158	–0.236	0.078	0.394	0.346	0.641
	Br_2	2.970	2.470	6.4%	1.074	–0.116	–0.214	0.098	0.330	0.312	0.692
	I_2	3.193	2.805	4.4%	1.022	–0.080	–0.194	0.114	0.274	0.274	0.741
	IBr	3.122	2.633	5.2%	1.034	0.004	–0.311	0.315	0.307	0.335	0.691
	ICl	3.064	2.494	6.0%	1.043	0.076	–0.411	0.487	0.335	0.393	0.632
N	Cl_2	2.411	2.100	4.4%	–0.477	–0.011	–0.148	0.137	0.159	0.169	0.852
	Br_2	2.472	2.405	3.6%	–0.491	0.011	–0.178	0.188	0.166	0.184	0.836
	I_2	2.648	2.759	2.7%	–0.511	0.036	–0.177	0.213	0.141	0.162	0.856
	IBr	2.569	2.587	3.4%	–0.514	0.139	–0.301	0.440	0.162	0.203	0.800
	ICl	2.506	2.449	4.1%	–0.519	0.229	–0.405	0.634	0.176	0.241	0.733

Table S9. Total electronic energy (E_0, Hartree), Zero-Point Energy correction (ZPE, Hartree), sum of electronic and thermal enthalpy ($E_0+\Delta H$, Hartree), sum of electronic and thermal free energy ($E_0+\Delta G$, Hartree), resulting formation enthalpy (ΔH_f, kcal/mol) and Gibbs free energy (ΔG_f, kcal/mol) calculated in the gas phase for **L** and corresponding adducts.

Compound	Type	E_0	ZPE	E_0+ZPE	$E_0+\Delta H$	$E_0+\Delta G$	ΔH_f	ΔG_f
**L**		-656.458	0.205	-656.253	-656.238	-656.296		
Cl_2		-29.897	0.001	-29.896	-29.893	-29.918		
Br_2		-26.358	0.001	-26.358	-26.354	-26.382		
I_2		-22.820	0.000	-22.819	-22.815	-22.845		
ICl		-26.363	0.001	-26.362	-26.358	-26.386		
IBr		-24.590	0.001	-24.590	-24.586	-24.615		
**L**·Cl_2	N-CT	-686.363	0.207586	-686.156	-686.137	-686.207	-3.704	4.681
**L**·Br_2	N-CT	-682.827	0.207	-682.620	-682.601	-682.673	-5.463	3.286
**L**·I_2	N-CT	-679.288	0.207	-679.081	-679.062	-679.135	-5.104	3.774
**L**·ICl	N-CT	-682.840	0.207	-682.633	-682.614	-682.684	-10.868	-1.076
**L**·IBr	N-CT	-681.063	0.207	-680.856	-680.837	-680.909	-7.999	1.583
**L**·Cl_2	Te-CT	-686.368	0.207	-686.161	-686.142	-686.212	-7.048	1.115
**L**·Br_2	Te-CT	-682.832	0.207	-682.625	-682.606	-682.678	-8.333	-0.167
**L**·I_2	Te-CT	-679.292	0.206324	-679.086	-679.066	-679.141	-7.794	0.243
**L**·ICl	Te-CT	-682.843	0.207	-682.636	-682.617	-682.688	-12.427	-3.482
**L**·IBr	Te-CT	-681.066	0.207	-680.860	-680.840	-680.913	-10.122	-1.419
**L**Cl_2	seesaw	-686.422	0.208	-686.214	-686.195	-686.263	-40.396	-30.485
**L**Br_2	seesaw	-682.858	0.207	-682.651	-682.632	-682.702	-24.760	-14.932
**L**I_2	seesaw	-679.298	0.207	-679.091	-679.072	-679.144	-11.537	-1.829
**L**ICl	seesaw	-682.859	0.207	-682.652	-682.633	-682.703	-22.904	-12.641
**L**IBr	seesaw	-681.078	0.207	-680.871	-680.852	-680.923	-17.346	-7.141

Table S10. Selected experimental and calculated structural metric parameters, energies, and Raman shift values for compound **L**I_2 (**1**) considered for the validation of the computational setup.

	Experim.	mPW1PW		PBE0		M06-2X		PBE0-D3	
Def2 BS		SVP	TZVP	SVP	TZVP	SVP	TZVP	SVP	TZVP
Te–I / Å	2.9092(4)[a]	2.927	2.923	2.921	2.917	2.913	2.909	2.915	2.911
N···Te / Å	2.97(1)[a]	2.946	2.963	2.943	2.961	2.975	2.994	2.930	2.948
I–Te–I / °	174.244(8)	174.78	174.86	174.42	174.47	173.60	173.39	173.24	173.28
ν_{sym} / cm^{-1}	110	115.3	115.9	116.3	117.0	120.1	120.9	118.2	118.8
ν_{asym} / cm^{-1}	–	187.0	184.1	187.0	184.2	188.6	185.6	186.9	184.2
E_0 (Ha)	–	-679.298	-679.986	-678.661	-679.345	-678.991	-679.704	-678.684	-679.367
$E_0+\Delta G$ (Ha)	–	-679.144	-679.833	-678.507	-679.192	-678.836	-679.551	-678.530	-679.215

[a] Mean value.

Table S11. Selected calculated structural and spectroscopic structural metric parameters, energies, and Raman shift values for the model CT N-adduct **L**·I_2 considered for the validation of the computational setup.

	mPW1PW		PBE0		M06-2X		PBE0-D3	
Def2 BS	SVP	TZVP	SVP	TZVP	SVP	TZVP	SVP	TZVP
N···I / Å	2.648	2.660	2.657	2.650	2.674	2.697	2.629	2.636
I–I / Å	2.759	2.754	2.752	2.753	2.743	2.737	2.758	2.754
N···I–I / °	174.63	174.47	174.49	174.45	174.63	174.48	175.19	175.07
Te–C–C–N / °	144.49	146.26	34.24	146.60	142.62	143.74	141.39	142.91
$\nu_{I\text{–}I}$ / cm^{-1}	183.6	183.7	185.4	184.4	188.0	188.7	184.3	184.1
E_{CT} / kcal/mol	22.80	20.33	22.09	20.92	22.96	20.01	24.06	21.75
E_0 (Ha)	-679.287	-679.974	-678.650	-679.333	-678.977	-679.689	-678.669	-679.351
$E_0+\Delta G$ (Ha)	-679.135	-679.822	-678.498	-679.181	-678.824	-679.537	-678.516	-679.200
